# Supplementary material for: Association between homicide rates and suicide rates: a countrywide longitudinal analysis of 5507 Brazilian municipalities
Source: BMJ Open. 2020 Nov 4;10(11):e040069. doi: 10.1136/bmjopen-2020-040069 (PMC7643512; doi:10.1136/bmjopen-2020-040069)
Supplement: Supplementary data [file bmjopen-2020-040069supp001.pdf]

Supplementary Material

Machado DB, McDonald K, Castro-de-Araujo LFS, Devakumar D, Alves FJO, et al. **The association between homicide rates and suicide rates: a countrywide longitudinal analysis of 5,507 Brazilian municipalities.**

Contents

1. **Supplemental Methods**. Additional details about CAPS coverage
2. **Supplemental Figure 1.** Distribution of age-standardised suicide rates per 100,000.
3. **Supplemental Table 1.** Unadjusted and adjusted associations between age-standardized suicide and age-standardised homicide rates in all Brazilian municipalities, only municipalities with accurate vital information in Brazil, and by population size, 2008-2014.
4. **Supplemental Table 2**. Unadjusted and adjusted associations between suicide rates and homicide rates in all Brazilian municipalities for 2010 only.
5. **Supplemental References**

Supplemental Methods: Additional details about CAPS coverage

The Psychosocial Community Centers (CAPS) were developed to provide day hospital care severe mental disorders. There are five different types of CAPS, classified based on their complexity, population coverage, and funding. CAPS I are based in small towns (20,000-50,000 inhabitants), CAPS II are based in mid-sized cities (50,000 or more inhabitants), and CAPS III are large units intended to provide 24-hour services. CAPSi provide services to children and adolescents. CAPSad are specialised units to treat problems associated with drug and alcohol use. The Brazilian government estimates CAPS coverage within each municipality according to the proportion of the population served by the different types of units (1,2). Specifically, this is calculated as:

{[(number of CAPS I x 0.5) + (number of CAPS II) + (number of CAPS III x 1.5) + (number of CAPSi) + (number of CAPSad)] x 100,000} / municipal population

Supplemental Figure 1. Distribution of age-standardised suicide rates per 100,000.

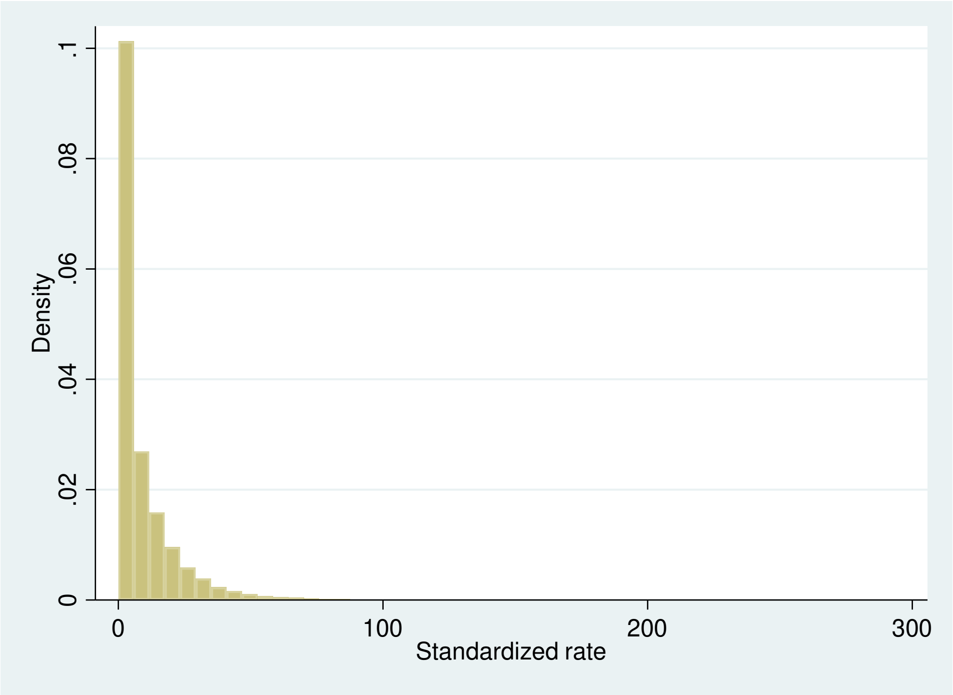

Supplemental Table 1. Unadjusted and adjusted associations between age-standardized suicide and age-standardised homicide rates in all Brazilian municipalities, only municipalities with accurate vital information in Brazil, and by population size, 2008-2014.

	Unadjusted model		Adjusted model[a]			
	RR[b]	95% CI	RR[b]	95% CI	Number of suicides	Number of municipalities[c]
All Brazilian municipalities	1.27	1.17-1.38	1.24	1.14-1.35	35,357	5,051
Municipalities with accurate vital information	1.46	1.23-1.72	1.45	1.22-1.72	9,336	1,556
Population <=10,000	1.21	1.03-1.41	1.21	1.03-1.41	14,352	2,100
Population 10,001-50,000	1.30	1.14-1.48	1.28	1.12-1.46	16,583	2,443
Population >50,000	1.33	1.15-1.55	1.32	1.13-1.54	4,259	639

Abbreviations: RR=Rate ratio; CI=Confidence interval.
[a] Models adjusted for monthly per capita income (BR$), CAPS coverage, urbanisation, percentage of people unemployed, percentage of individuals who were divorced, percentage of Pentecostal Christians, and percentage of households with one resident.
[b] Change in suicide rate per 100% increase in homicide rate.
[c] This number excludes municipalities with zero suicides recorded between 2008 and 2014.

Machado DB, *et al. BMJ Open* 2020; 10:e040069. doi: 10.1136/bmjopen-2020-040069

Supplemental Table 2. Unadjusted and adjusted associations between suicide rates and homicide rates in all Brazilian municipalities for 2010 only.

	Unadjusted model		Adjusted model[a]			
**Year**	**RR[b]**	**95% CI**	**RR[b]**	**95% CI**	**Number of suicides**	**Number of municipalities[c]**
2010	0.51	0.46-0.64	0.91	0.77-1.08	9287	5,506

Abbreviations: RR=Rate ratio; CI=Confidence interval.
[a] Models adjusted for monthly per capita income (BR$), CAPS coverage, urbanisation, percentage of people unemployed, percentage of individuals who were divorced, percentage of Pentecostal Christians, and percentage of households with one resident.
[b] Change in suicide rate per 100% increase in homicide rate.
[c] This number excludes municipalities with zero suicides recorded in 2010.

Machado DB, *et al. BMJ Open* 2020; 10:e040069. doi: 10.1136/bmjopen-2020-040069

Supplemental References

1. Ministério da Saúde. Portaria GM/MS 336 [Internet]. Ministério da Saúde, editor. 2002. Available from: http://www.saude.mg.gov.br/images/documentos/Portaria_336.pdf
2. Ministério da Saúde. Portaria GM/MS 2.669 [Internet]. Ministério da Saúde, editor. 2009. Available from: http://bvsms.saude.gov.br/bvs/saudelegis/gm/2009/prt2669_03_11_2009.html
