# Supplementary material for: Association between homicide rates and suicide rates: a countrywide longitudinal analysis of 5507 Brazilian municipalities
Source: BMJ Open. 2020 Nov 4;10(11):e040069. doi: 10.1136/bmjopen-2020-040069 (PMC7643512; doi:10.1136/bmjopen-2020-040069)
Supplement: Author's manuscript [file bmjopen-2020-040069.draft_revisions.pdf]

BMJ Open

BMJ Open is committed to open peer review. As part of this commitment we make the peer review history of every article we publish publicly available.

When an article is published we post the peer reviewers' comments and the authors' responses online. We also post the versions of the paper that were used during peer review. These are the versions that the peer review comments apply to.

The versions of the paper that follow are the versions that were submitted during the peer review process. They are not the versions of record or the final published versions. They should not be cited or distributed as the published version of this manuscript.

BMJ Open is an open access journal and the full, final, typeset and author-corrected version of record of the manuscript is available on our site with no access controls, subscription charges or pay-per-view fees (http://bmjopen.bmj.com).

If you have any questions on BMJ Open's open peer review process please email info.bmjopen@bmj.com

BMJ Open

The association between homicide rates and suicide rates: a countrywide longitudinal analysis of 5,507 Brazilian municipalities

Journal:	*BMJ Open*
Manuscript ID	bmjopen-2020-040069
Article Type:	Original research
Date Submitted by the Author:	04-May-2020

Complete List of Authors:	Machado, Daiane Borges; Center of Data and Knowledge Integration for Health<br>McDonald, Keltie; University College London, Division of Psychiatry<br>Araújo , Luis ; Center of Data and Knowledge Integration for Health<br>Devakumar, Delan; University College London Research Department of Epidemiology and Public Health,<br>Alves, Flávia; Center of Data and Knowledge Integration for Health<br>Kiss, Lígia; University College London, Institute for Global Health<br>Lewis, Glyn; University College London, Division of Psychiatry<br>Barreto, Mauricio; Federal University of Bahia, Instituto de Saúde Coletiva

Keywords:	EPIDEMIOLOGY, PUBLIC HEALTH, PSYCHIATRY, MENTAL HEALTH, Suicide & self-harm < PSYCHIATRY

SCHOLARONE™
Manuscripts

I, the Submitting Author has the right to grant and does grant on behalf of all authors of the Work (as defined in the below author licence), an exclusive licence and/or a non-exclusive licence for contributions from authors who are: i) UK Crown employees; ii) where BMJ has agreed a CC-BY licence shall apply, and/or iii) in accordance with the terms applicable for US Federal Government officers or employees acting as part of their official duties; on a worldwide, perpetual, irrevocable, royalty-free basis to BMJ Publishing Group Ltd ("BMJ") its licensees and where the relevant Journal is co-owned by BMJ to the co-owners of the Journal, to publish the Work in this journal and any other BMJ products and to exploit all rights, as set out in our licence.

The Submitting Author accepts and understands that any supply made under these terms is made by BMJ to the Submitting Author unless you are acting as an employee on behalf of your employer or a postgraduate student of an affiliated institution which is paying any applicable article publishing charge ("APC") for Open Access articles. Where the Submitting Author wishes to make the Work available on an Open Access basis (and intends to pay the relevant APC), the terms of reuse of such Open Access shall be governed by a Creative Commons licence – details of these licences and which Creative Commons licence will apply to this Work are set out in our licence referred to above.

Other than as permitted in any relevant BMJ Author's Self Archiving Policies, I confirm this Work has not been accepted for publication elsewhere, is not being considered for publication elsewhere and does not duplicate material already published. I confirm all authors consent to publication of this Work and authorise the granting of this licence.

For peer review only

The association between homicide rates and suicide rates: a countrywide longitudinal analysis of 5,507 Brazilian municipalities

Daiane Borges Machado[1,2]*, Keltie McDonald[3]*, Luís F S Castro-de-Araujo[1,4], Delanjathan Devakumar[5], Flávia Jôse Oliveira Alves[1], Lígia Kiss[5], Glyn Lewis[3], Maurício L. Barreto[1,6].

*Denotes equal contribution as first author

Corresponding author: Keltie McDonald; k.mcdonald@ucl.ac.uk; Division of Psychiatry, University College London, 6th Floor Maple House, 149 Tottenham Court Road, London, W1T 7NF, UK.

[1] Center of Data and Knowledge Integration for Health (CIDACS), Salvador, Brazil.

[2] Centre for Global Mental Health, London School of Hygiene & Tropical Medicine (LSHTM).

[3] Division of Psychiatry, University College London, London, United Kingdom.

[4] The University of Melbourne, Department of Psychiatry, Austin Health, Heidelberg, Victoria, Australia.

[5] Institute for Global Health, University College London, London, United Kingdom.

[6] Institute of Collective Health, Federal University of Bahia, Salvador, Brazil.

Keywords: homicide, suicide, ecological study, longitudinal study, epidemiology, public health, mental health

Word count: 3,987

For peer review only - http://bmjopen.bmj.com/site/about/guidelines.xhtml

ABSTRACT

Objective: To estimate the association between homicide rates and suicide rates in Brazilian municipalities over a period of 7 years.

Design: We conducted a longitudinal ecological study using annual mortality data from 5,507 Brazilian municipalities between 2008 and 2014. Multivariable negative binomial regression models were used to examine the relationship between homicide and suicide rates. Robustness of results was explored using sensitivity analyses to examine the influence of data quality, population size, age, and sex on the relationship between homicide and suicide rates.

Setting: A nationwide study of municipalities-level data.

Participants: Mortality data and corresponding population estimates for municipal populations aged 10 years and older.

Primary and Secondary Outcome Measures: Age-standardised suicide rates per 100,000.

Results: Municipal suicide rates were positively associated with municipal homicide rates; after adjusting for socioeconomic and demographic factors, a doubling of the homicide rate was associated with 22% increase in suicide rate (RR=1.22, 95% CI 1.26-1.33). A dose-response effect was observed with 4% increase in suicide rates at the third quintile, 9% at the fourth quintile and 12% at the highest quintile of homicide rates compared with the lowest quintile. The observed effect estimates were robust to sensitivity analyses.

Conclusions: Municipalities with higher homicide rates have higher suicide rates, and the relationship between homicide and suicide rates in Brazil exists independently of many sociodemographic and socioeconomic factors. The identification of a positive relationship between homicide and suicide rates suggests that population-based

For peer review only - http://bmjopen.bmj.com/site/about/guidelines.xhtml

interventions to reduce homicide rates may also reduce suicide rates in Brazil.

For peer review only

For peer review only - http://bmjopen.bmj.com/site/about/guidelines.xhtml

STRENGTHS AND LIMITATIONS OF THIS STUDY

- In this longitudinal ecological study, we examined the association between homicide and suicide rates using data from 5,507 municipalities in Brazil from 2008 to 2014, including several sensitivity analyses to address limitations of previous examinations of this topic.
- Homicide and suicide are important public health concerns in Brazil and globally, and our findings provide clear evidence for their relationship, and suggest potential avenues for public health and policy interventions to reduce deaths from these causes.
- Our study used an ecological design, and therefore, results are generalisable to the municipal population-level and not to individuals.

For peer review only

For peer review only - http://bmjopen.bmj.com/site/about/guidelines.xhtml

KEY QUESTIONS

What is already known?

Existing studies of the relationship between homicide rates of suicide rates have primarily examined mortality data from large geographical regions such as countries or multi-country regions. Evidence suggests considerable regional variations and conflicting evidence for the magnitude and direction of the relationship. Very few studies examined the influence of possible confounding factors such as socioeconomic or demographic features on the relationship at the population-level.

What are the new findings?

In this longitudinal ecological study, we examined the association between homicide and suicide rates using data from 5,507 municipalities in Brazil from 2008 to 2014. We observed that areas with higher homicide rates showed significantly increased suicide rates. Finds were robust to several sensitivity analyses.

What do the new findings imply?

Understanding the relationship between homicide and suicide rates in the population may help to identify avenues for public health interventions to reduce mortality due to these causes. The relationship between homicide and suicide appears to be complex, with strong geographical variations, likely due to the interaction of local social, cultural, and economic factors. The findings from this study suggest that, in Brazil, a positive relationship between homicide and suicide rates persists independently of many of these factors.

For peer review only

For peer review only - http://bmjopen.bmj.com/site/about/guidelines.xhtml

INTRODUCTION

Suicide is an important global public health problem, causing over 800,000 deaths per year. Amongst 15-29 year-olds, suicide is the second leading cause of mortality worldwide (1). One of the strongest and most consistently identified risk factors for suicide is psychiatric disorder (2). In Brazil in 2012, the national rate of suicide was 6.2 per 100,000 inhabitants, and was higher in males (10.0 per 100,000) than females (2.7 per 100,000) (3). The rate of suicide has increased in nearly all age groups for males and females between 2000 and 2016 (4).

In 2008, the World Health Organization (WHO) reported violence as a leading cause of death and disability and a "global challenge" (1). Violence is not exclusively a criminal justice issue; it is also a health and human rights issue. Over 90% of deaths caused by violence occur in low and middle income countries (LMICs), however, most studies into violent deaths have been carried out in high-income countries (HICs) (5). In Brazil, the homicide rate has increased over the last three decades (6). In 2016, interpersonal violence was the second leading cause of age-standardised years of life lost in Brazil (7).

Brazil is the largest country in Latin America with 207 million inhabitants. Despite, being the 9th richest country in the world in terms of nominal Gross Domestic Product, Brazil has one of the highest levels of income inequality in the world (8,9). Homicide in Brazil has been related to socioeconomic inequalities (6,10,11), poverty (12), race, gender and age, with men, younger people, and those identifying as black at higher risk

For peer review only - http://bmjopen.bmj.com/site/about/guidelines.xhtml

(6,10), while suicide has also been associated with income inequality, sex, and age and low levels of education (3,4).

While often studied separately, there is a long tradition of viewing homicide and suicide as related phenomena (13). Homicide and suicide can be viewed similarly as forms of violence (1). Several studies have examined the relationship between homicide and suicide rates in the population; however, evidence is mixed (see Research in Context panel). There appear to be strong variations in the magnitude and direction of association across countries (14,15). Many of the previous studies of the association have been conducted in HICs. However, the relationship between homicide and suicide rates in LMICs might be different due to different distributions and influences of risk factors in the population. There has been only one previous study of the ecological association between homicide and suicide rates in Brazil, which observed an inverse correlation in 2010 (16). However, this study used data from a single year, studied large, heterogeneous geographical areas (27 Brazilian states), and did not adjust for important demographic and socioeconomic factors. We aimed to further investigate the possible association between homicide and suicide rates in Brazil, using a dataset of all Brazilian municipalities from 2008 to 2014.

For peer review only

METHODS

Study Design

This study used a longitudinal ecological design, using yearly data from the 5,507 Brazilian municipalities for the period 2008 to 2014.

Data Sources

All data used in this study are publicly available online (http://www2.datasus.gov.br). To estimate suicide and homicide rates, cause-specific mortality data were collected from the Brazilian Ministry of Health's Mortality Information System. All deaths are recorded in this system using the International Classification of Diseases, 10th revision (17). Socioeconomic, demographic variables (monthly per capita income in Brazilian Real (BR$); unemployment rate; percentage of individuals who were divorced; percentage of individuals who declared being Pentecostal; percentage of households with only one resident; and urbanisation), and municipality population estimates were obtained from the Brazilian Institute of Geography and Statistics (IBGE). Data from the main mental health services in Brazil, the Psychosocial Care Centre (CAPS) coverage was obtained from the mental health coordination database, held by the Ministry of Health (DATASUS).

Definition of variables

Outcome

Suicide is defined as a death resulting from intentional self-harm according to the International Classification of Diseases, 10th revision (ICD-10) codes X60 to X84 (17). In Brazil, all deaths due to external causes (suicide, homicide, and accidents) are forwarded to the Medical Legal Institute (IML; artigo 2º da Resolução CFM nº. 1.779/2005) where the death certificates are printed and signed by an examining doctor (18), which decreases the chances for misclassification. Diagnoses are based on an autopsy (using the ICD-10 classification), to investigate the circumstances in which the death occurred, personal history of the victim, and suicide risk factors (19).

For peer review only - http://bmjopen.bmj.com/site/about/guidelines.xhtml

The suicide rate was calculated at the municipality level and as it is influenced by age, we standardized by each 5-year age range, from 10 years or older. We used direct standardisation using the WHO population distribution as the standard population. Suicides among individuals under 10 years-old were excluded because suicide is a very rare event before 10 years; there were 33 victims of suicide under 10 years in Brazil in the period 2008-2014 (http://www2.datasus.gov.br). Overall and sex-specific age-standardised suicide rates were calculated for each municipality and year of analysis, expressed as a rate per 100,000 inhabitants.

Exposure

Homicide was defined as a death resulting from injuries inflicted by another person with the intent to injure or kill using any means according to the ICD-10 codes X85 to Y09 (17). Homicide rates were also calculated at the municipality level. Homicide rates were not standardized by age because it was used as proxy for violence, and therefore, total violence irrespective of age distribution was of interest. Overall and sex-specific homicide rates were calculated for each municipality and year of analysis, expressed as a rate per 100,000 inhabitants.

Confounders

Municipality-level covariates for suicide considered in the model included: the monthly per capita income in Brazilian Real (BR$); unemployment rate, measured by the percentage of the population aged 16 years or over with no official job; percentage of individuals who were divorced; percentage of individuals who declared being Pentecostal; percentage of households with only one resident; and urbanisation,

For peer review only - http://bmjopen.bmj.com/site/about/guidelines.xhtml

measured as the percentage of individuals living in urban areas for each municipality. These variables were selected based on evidence in the literature that they may be associated with suicide (3,20–22).

We also included the Psychosocial Care Centre (CAPS) coverage, defined as the proportion of people who could potentially be seen in the centres, because availability of mental health care may be associated with suicide rates. CAPS coverage was calculated for each municipality based on the different types of CAPS available and the populations that they are expected to serve (Supplemental Methods).

Statistical Analysis

We used negative binomial regression analysis to evaluate the association between age-standardized suicide rates and rates of homicide. Year of study (a time-specific fixed effect) was introduced into the models to control for the national-level policy changes or secular trends that may have affected all municipalities (23).

The main estimated regression model was: $\log(Y_{it}) = \alpha_i + \beta_1 HOMICIDE_{it} + \beta_n X_{nit} + \gamma_t + u_{it}$, where t =year and i =municipality, Y_{it} is the standardized mortality rate (per 100,000 inhabitants) due to suicide, α_i is the fixed effect for municipality i capturing unobserved time-invariant factors causing between-municipality variability in suicide, $HOMICIDE_{it}$ was the rate of homicide for the municipality i in the year t, X_{nit} is the value taken by a covariate vector containing the n socioeconomic and demographic determinants included in the model, for municipality i in year t, γ_t is a time-specific effect, capturing variability in suicide rates over time, u_{it} is an error term, assumed to follow a normal

For peer review only - http://bmjopen.bmj.com/site/about/guidelines.xhtml

distribution. To produce rate ratio (RR) estimates that were more readily interpretable, the homicide rate was divided by 100 (i.e. homicide rate per 1,000).

The age-standardised suicide rate, although not a count, still appeared to follow a binomial distribution (Supplemental Figure 1). We compared a negative binomial model to a Poisson regression model using an Akaike's Information Criterion and the Bayesian Information Criterion to establish the model that best fitted the data (24). We found evidence that the age-standardised suicide rates varied between municipalities more than expected under a Poisson distribution, therefore, the negative binomial model was used to allowed for overdispersion. The Hausman test was used to compare a negative binomial regression with fixed effects to a model with a random effect for each municipality. The test showed no evidence that the random effects model provided a better fit to the data (p=0.05), therefore, our model included only fixed effects.

Population standardization was used to allow comparability across municipalities with distinct age structures. We also tested the association using models based on both age-standard homicide rates and age-standardised suicide rates, and the results were similar (Supplemental Table 1).

Primary Analysis

To test the association between homicide and age-standardised suicide rate we constructed a negative binomial regression model including data from all Brazilian municipalities. We produced both unadjusted models and models adjusting for confounders.

For peer review only - http://bmjopen.bmj.com/site/about/guidelines.xhtml

Secondary Analyses

To examine the robustness of our primary findings, we fitted several additional negative binomial regression models: one including only municipalities considered to have very accurate vital information according to a previous study (25), three by total municipal population (10,000 inhabitants; 10,001-50,000; and more than 50,000 inhabitants), one by municipal homicide rates quintiles, and six further stratified by sex and age groups (10-19 years; 20-59 years; 60 and older). Finally, we tested the association between homicide rates and age-standardised suicide rates for 2010 only to enable a comparison with the results of a previous study of Brazilian states (16). For each analysis, we fitted both unadjusted and adjusted models. Statistical analyses were performed using Stata 15 software (26).

Patient and Public Involvement

There was no patient and public involvement in this study.

RESULTS

The age-standardised suicide rate increased in the Brazilian municipalities from 7.6/100,000 in 2008 to 8.2/100,000 inhabitants in 2014. Homicide rates also increased over this period, from 15.2/100,000 to 19.6/100,000 inhabitants (**Figure 1**). Unemployment decreased between 2008 and 2014, while all other covariates increased (**Table 1**).

For peer review only

Table 1. Mean values and standard deviations of suicide and homicide rates and selected covariates for the Brazilian municipalities (n=5,507) in 2008 and 2014.

	2008		2014		
**Outcome**	**Mean**	**SD**	**Mean**	**SD**	**Percentage Change[c]**
Suicide rate[a,b]	7.58	12.79	8.18	13.25	7.97
Homicide rate[b]	15.21	18.39	19.65	21.84	29.16
CAPS coverage per municipal population	0.26	0.73	0.45	0.97	72.13
Monthly per capita income (BR$)	453.30	227.58	540.53	265.35	19.24
Unemployed people (%)	7.15	3.72	4.85	4.22	-32.16
Individuals who were divorced (%)	3.37	1.75	4.13	2.10	22.73
Pentecostals Christians (%)	10.43	6.17	12.10	7.15	15.96
Households with one resident (%)	11.02	2.88	12.82	3.33	16.27
Urbanisation (%)	63.04	22.03	66.26	21.80	5.10

Abbreviations: SD=standard deviation.
[a] Age-standardised.
[b] Rate per 100,000.
[c] Percentage change from 2008.

We found a positive association between homicide rates and age-standardised suicide rates in the unadjusted model and after controlling for confounders (**Table 2**). In the model based on all Brazilian municipalities, a doubling of the homicide rate was associated with 22% increase in the suicide rate (RR=1.22, 95% CI: 1.13-1.33). We found similar results in the models stratified by municipal population sizes. Sensitivity analyses, based on a subset of municipalities considered to have accurate data, yield similar results but with a stronger association (RR=1.44, 95% CI: 1.22-1.70).

For peer review only - http://bmjopen.bmj.com/site/about/guidelines.xhtml

Table 2. Unadjusted and adjusted associations between suicide rates and homicide rates in all the Brazilian municipalities, only municipalities with accurate vital information in Brazil, and by population size, 2008-2014.

	**Unadjusted model**		**Adjusted model[a]**			
	**RR[b]**	**95% CI**	**RR[b]**	**95% CI**	**Number of suicides**	**Number of municipalities[c]**
All Brazilian municipalities	1.25	1.16 to 1.36	1.22	1.13 to 1.33	35,357	5,051
Municipalities with accurate vital information	1.45	1.23 to 1.70	1.44	1.22 to 1.70	9,336	1,556
Population ≤10,000	1.19	1.01 to 1.39	1.18	1.01 to 1.39	14,352	2,100
Population 10,001-50,000	1.29	1.13 to 1.47	1.27	1.11 to 1.45	16,583	2,443
Population >50,000	1.29	1.12 to 1.49	1.28	1.10 to 1.48	4,259	639

Abbreviations: CI=confidence interval; RR=rate ratio.
[a] Model adjusted for monthly per capita income (BR$), CAPS coverage, urbanisation, percentage of people unemployed, percentage of individuals who were divorced, percentage of Pentecostal Christians, and percentage of households with one resident.
[b] Change in suicide rate per 100% increase in homicide rate.
[c] This number excludes municipalities with zero suicides recorded between 2008 and 2014.

For peer review only - http://bmjopen.bmj.com/site/about/guidelines.xhtml

Analysing by quintile of homicide rate showed that the higher the homicide rate, the stronger the association, with 1% suicide rate increase at the second quintile, 4% increase at the third quintile, 9% increase at the fourth quintile, and 12% at the highest quintile compared with those in the lowest quintile (**Table 3**). The analysis stratified by sex and age groups showed a positive association between homicide rates and suicide among men from 20 to 59 years old (**Table 4**).

For peer review only

Table 3. Unadjusted and adjusted associations between suicide rates and homicide rates in the Brazilian municipalities by quintiles of homicide rates, 2008–2014[a]

	Unadjusted model		Adjusted model[b]	
**Homicide rate**	**RR[c]**	**95% CI**	**RR[c]**	**95% CI**
1st quintile (reference)	1		1	
2nd quintile	1.01	0.96 to 1.06	1.01	0.96 to 1.06
3rd quintile	1.04	0.99 to 1.09	1.04	0.99 to 1.09
4th quintile	1.10	1.04 to 1.15	1.09	1.04 to 1. .14
5th quintile	1.13	1.07 to 1.19	1.12	1.06 to 1.18

Abbreviations: CI=confidence interval; RR=rate ratio.

[a] Based on 35,357 suicides from 5,051 municipalities. Municipalities with zero suicides recorded between 2008 and 2014 were excluded.

[b] Model adjusted for monthly per capita income (BR$), CAPS coverage, urbanisation, percentage of unemployed people, percentage of individuals who were divorced, percentage of Pentecostal Christians, and percentage of households with one resident.

[c] Change in suicide rate per 100% increase in homicide rate.

For peer review only - http://bmjopen.bmj.com/site/about/guidelines.xhtml

Table 4. Unadjusted and adjusted associations between suicide rates and homicide rates in the Brazilian municipalities, by sex and age groups, 2008–2014.

		Unadjusted model		Adjusted model[a]			
**Age range**	**Sex**	**RR[b]**	**95% CI**	**RR[b]**	**95% CI**	**Number of suicides**	**Number of municipalities[c]**
10-19	Men	1.24	0.91-1.71	1.20	0.87-1.66	10,626	1,518
	Women	1.53	0.96-2.42	1.53	0.96-2.43	6,167	881
20-59	Men	1.29	1.17-1.43	1.24	1.12-1.38	32,382	4,626
	Women	1.23	1.00-1.51	1.20	0.98-1.47	19,187	2,741
60 and older	Men	1.12	0.90-1.39	1.05	0.84-1.32	19,096	2,728
	Women	1.39	0.89-2.18	1.28	0.80-2.01	7,455	1,065

Abbreviations: CI=confidence interval; RR=rate ratio.
[a] Model adjusted for monthly per capita income (BR$), CAPS coverage, urbanisation, percentage of unemployed people, percentage of individuals who were divorced, percentage of Pentecostal Christians, and percentage of households with one resident.
[b] Change in suicide rate per 100% increase in homicide rate.
[c] This number excludes municipalities with zero suicides recorded between 2008 and 2014.

review only

We repeated the analysis based on data from 2010 only. The unadjusted analysis showed a significant inverse relationship between homicide rates and suicide rates in 2010 (RR=0.51, 95% CI: 0.746-0.64). However, after adjusting for confounders the estimate of association became closer to the null value (RR=1.0) and was longer significant (RR=0.91, 95% CI: 0.77-1.08; Supplemental Table 2).

DISCUSSION

Main Findings

We assessed the relationship between homicide and suicide rates in Brazilian municipalities over a 7-year period. We found that municipalities with higher homicide rates also had higher age-standardised suicide rates in both unadjusted and adjusted models. Our results appeared to be robust based on several sensitivity analyses. Similar results were observed for models stratified by population size, and for models including only municipalities considered to have accurate data (25). The association between homicide and suicide rates seemed to follow a dose-response pattern, such that areas with higher homicide rates showed stronger relationships with increased suicide rates, reaching 12% increase in the highest quintile of homicide rates compared with the lowest.

Strengths and Limitations

This study examined the relationship between homicide and suicide rates in detail. Our data covered all 5,507 municipalities in Brazil and are, therefore, representative of the country. The use of fine-grained geographical areas likely resulted in less within-area heterogeneity and provided improved opportunity to control for confounding factors.

For peer review only - http://bmjopen.bmj.com/site/about/guidelines.xhtml

The estimates of association observed in this study appear to be robust, with minimal changes in magnitude between models accounting for different potential sources of bias. Some limitations of this study are acknowledged. This study used an ecological design, and therefore conclusions are applicable to populations rather than individuals. An ecological design was used due to data availability, and only the use of aggregate data allows the study of the total Brazilian population longitudinally. Furthermore, given our interest in homicide rates as an indicator of contextual violence, aggregate data for homicide were required.

Another limitation could be that large municipalities and small municipalities have equal weight in the analysis. For this reason, we have also provided estimates after stratification by municipal population size.

Potential bias resulting from misclassification of suicides and homicides is another possible limitation. Homicide and suicide deaths may have a risk of misclassification as events of undetermined intent or unspecified accidents. Suicide may be underreported due to stigmatisation and social taboo, while homicides could be under-reported as they may indicate unrest or instability. It is unclear whether the frequency of misclassification differs between homicides and suicides. If misclassification of homicides and suicides occurred equally frequently, then the observed estimates of association between homicide and suicide rates are conservative. In fact, the observed estimate of association was slightly stronger when only municipalities judged to have high-quality vital information were included. Nevertheless, misclassification is not expected to be a major concern because all deaths due to reported external causes, including homicides and suicides, are determined based on autopsy (19).

For peer review only - http://bmjopen.bmj.com/site/about/guidelines.xhtml

Results in Context

The results of this study are in contrast with a previous cross-sectional ecological study of the association between homicide and suicide rates in Brazil, which observed a significant inverse correlation between the two forms of death. In their study, Bando and Lester found a correlation of -0.61 ($p<0.001$) between age-standardised homicide rates and age-standardised suicide rates in 27 Brazilian states, and after stratification by sex, the correlation remained significant in men, but not women (16). Our unadjusted analysis using only the 2010 data showed similar results to those observed by Bando and Lester, however, after adjusting for confounders the association was no longer significant. Confounding factors appeared to have considerable influence on the observed relationship, and the inverse association in the unadjusted analysis is likely a reflection of bias. Further, it is notable that data for our analysis were aggregated to the municipality-level (n=5507), rather than state-level (n=27). Using more fine-grained geographical areas likely resulted in less heterogeneity within regions in terms of important determinants of homicide and suicide.

Our study found some differences in the association by age group. We found no association between increased homicide rates with increased suicide among those 10 to 19 years-old or 60 years and older. However, a statistically significant association was observed among men and women 20 to 59 years old. It is possible that the limited number of suicides among the youngest and oldest age-sex stratifications resulted in insufficient power to achieve significance. Nevertheless, this observation is consistent with findings from a study of the correlations between homicide and suicide rates in Canada and US, which also showed the strongest correlations among 35-44 and 45-54

For peer review only - http://bmjopen.bmj.com/site/about/guidelines.xhtml

year-olds (27). Interestingly, there was nearly no difference in the strength of association between men and women within this age group, while both suicide and homicide rates tend to be higher in men (4,6).

In the broader literature, existing studies of the relationship between homicide and suicide rates provide mixed evidence. While some studies have observed positive relationships between homicide and suicide (15,27–29), others have observed an inverse relationship (30,31). Evidence based on multi-national data suggests differential relationships by country or geographical area in terms of both strength and direction of association (14,15). While a positive relationship between homicide and suicide is often observed in Europe (14,15,29), the Americas and Asian Pacific region tend to show an inverse or no evidence of a relationship (14,15), although some evidence of a positive association within the US has been shown (27). Importantly, large areas, such as whole countries, likely contain considerable variability in the distributions of important demographic and socioeconomic factors that influence homicide and suicide rates. Many of these studies have examined the association with limited control for potential confounding factors, which may help to explain the variations in correlation estimates observed between studies. Therefore, better evidence is required in order to draw clearer conclusions about the association beyond Brazil.

The relationship between homicide and suicide in the population has been studied for centuries (13). Several theoretical frameworks for understanding a positive relationship between homicide and suicide rates suggest that the two forms of death may share some common social causes, yet no single theory appears to adequately explain the mechanism underlying this relationship. Statistically, a relationship may arise due to a

For peer review only - http://bmjopen.bmj.com/site/about/guidelines.xhtml

causal association between homicide and suicide rates or due to shared causal factors. For example, high homicide rates may cause high suicide rates indirectly through low social cohesion within the geographical area under study. Alternatively, low social cohesion may cause high homicide rates and high suicide rates independently, therefore producing a spurious relationship.

We observed that the relationship between homicide and suicide rates persisted even after adjusting for several important factors including per capita income, CAPS coverage, urbanisation, unemployment, single-person households, divorce, and religious affiliation. Therefore, we have evidence that, in Brazil, the relationship exists, even after accounting for these factors. Furthermore, two of these variables—single-person households, and the percentage of individuals within the municipality that are divorced—may contribute to social cohesion. Since the relationship persists after adjusting for these, it is unlikely that low social cohesion adequately explains the relationship between homicide and suicide rates within the Brazilian municipalities. Notably, low social cohesion is only one possible explanation and there may be many others. Taken together, this is consistent with a hypothesis that homicide rates lead to changes in suicide rates, though we cannot be confident that this reflects a causal association. The relationship is likely complex, involving the interaction of social, cultural, economic, and political factors.

Targets of homicide and suicide prevention often have highly divergent orientations; while homicide prevention often involves the criminal justice system, suicide prevention usually involves the mental health system and reducing availability of methods for suicide. In Brazil, suicide is expected to be prevented at the community

For peer review only - http://bmjopen.bmj.com/site/about/guidelines.xhtml

level of care. After the Psychiatric Reformation movement, mental health care in Brazil was officially reformulated in 1978, and the CAPS were created to provide mental health care in the community (32,33). However, increased CAPS coverage was not found to reduce total suicides in Brazilian municipalities between 2008 and 2012 (22). Likewise, to try to control violence the Brazilian justice system has increased incarceration, and as a result, Brazil has the fourth-largest prison population in the world (6), and homicide rates have increased between 2004 and 2012 (21). This highlights the importance of investigating new factors and potential targets for intervention and prevention that may be associated with suicide and homicide rates in the country.

The presence of a relationship between the two suggests the co-benefits of interventions to reduce homicide and suicide deaths. For example, extensive evidence from the US shows that restricting access to firearms has been shown to reduce firearm-related homicide and suicide (34). The correlation between firearm availability and suicide and homicide appears to extend to other countries (35). In Brazil, a nationwide anti-firearm legislation was passed in 2004. There is some evidence that this legislation reduced both firearm-related suicide and firearm-related homicide, although the evidence for a reduction in total homicide and suicide is less clear (36,37). Unfortunately, the disarmament law was repealed in 2019. Future research may seek to identify other interventions at the policy-level, which may help to reduce both homicide, and suicide rates in the population.

For peer review only - http://bmjopen.bmj.com/site/about/guidelines.xhtml

CONCLUSION

Our longitudinal ecological study showed a robust positive relationship between homicide and suicide rates in Brazilian municipalities. We showed a dose-response relationship such that areas of higher homicide rates showed greater increase in suicide rates. The results of this study contribute clear evidence for the relationship to a literature that has previously shown conflicting evidence. A positive relationship between homicide and suicide rates can have important implications for public health and policy interventions as approaches to reduce one of homicide or suicide rates may also help to alleviate rates of the other. This can have important implications for Brazil and other countries where levels of violence are considerable.

CONTRIBUTORS

DM had full access to the data and was responsible for the data analysis. DM and KM contributed equally to the preparation of the manuscript. All other authors contributed to the interpretation of results, provided critical review of the manuscript, and had final responsibility for the decision to submit for publication.

FUNDING

The study was in part funded by the Medical Research Council (MC_PC_MR/R018677/1) and Wellcome Trust (202912/Z/16/Z). This work was also supported by the National Institute for Health Research, University College London Hospital, Biomedical Research Centre. The funders were not involved in the design and conduct of the study; collection, management, analysis, and interpretation of the data; preparation, review, or approval of the manuscript; and decision to submit the manuscript for publication.

For peer review only - http://bmjopen.bmj.com/site/about/guidelines.xhtml

COMPETING INTERESTS

The authors report no competing interests.

PATIENT CONSENT

Consent was not required for this study, which involved secondary analysis of aggregated, anonymised data.

DATA SHARING

All data used in this study are publicly available online (http://www2.datasus.gov.br).

For peer review only

REFERENCES

1. World Health Organization. Preventing suicide: A global imperative. Geneva, Switzerland, Switzerland: World Health Organization; 2014.
2. Nock MK, Borges G, Bromet EJ, Cha CB, Kessler RC, Lee S. Suicide and suicidal behavior. Epidemiol Rev. 2008;30(1):133–54.
3. Machado DB, Rasella D, Dos Santos DN. Impact of income inequality and other social determinants on suicide rate in Brazil. PLoS One. 2015;10(4):e0124934.
4. Martini M, da Fonseca RC, de Sousa MH, de Azambuja Farias C, Cardoso T de A, Kunz M, et al. Age and sex trends for suicide in Brazil between 2000 and 2016. Soc Psychiatry Psychiatr Epidemiol [Internet]. 2019;54(7):857–60. Available from: https://doi.org/10.1007/s00127-019-01689-8
5. World Health Organization. Preventing violence and reducing its impact: How development agencies can help. Geneva: Switzerland: World Health Organization; 2008.
6. Murray J, de Castro Cerqueira DR, Kahn T. Crime and violence in Brazil: Systematic review of time trends, prevalence rates and risk factors. Aggress Violent Behav. 2013;18(5):471–83.
7. Marinho F, de Azeredo Passos VM, Carvalho Malta D, Barboza França E, Abreu DMX, Araújo VEM, et al. Burden of disease in Brazil, 1990–2016: a systematic subnational analysis for the Global Burden of Disease Study 2016. Lancet. 2018;392(10149):760–75.
8. World Bank. GDP ranking [Internet]. 2017. Available from: https://datacatalog.worldbank.org/
9. World Bank. GINI index [Internet]. 2016. Available from: https://data.worldbank.org/indicator/SI.POV.GINI

For peer review only - http://bmjopen.bmj.com/site/about/guidelines.xhtml

10. Araújo EM de, Costa M da CN, Oliveira NF de, Santana F dos S, Barreto ML, Hogan V, et al. Spatial distribution of mortality by homicide and social inequalities according to race/skin color in an intra-urban Brazilian space. Rev Bras Epidemiol. 2010;13(4):549–60.

11. Gawryszewski VP, Costa LS. Social inequality and homicide rates in Sao Paulo City, Brazil. Rev Saude Publica. 2005;39(2):191–7.

12. Pereira DVS, Mota CMM, Andresen MA. Social disorganization and homicide in Recife, Brazil. Int J Offender Ther Comp Criminol. 2017;61(14):1570–92.

13. Bills CB. The Relationship between Homicide and Suicide: A narrative and conceptual review of violent death. Int J Confl Violence. 2017;11:a400–a400.

14. Bills CB, Li G. Correlating homicide and suicide. Int J Epidemiol. 2005;34(4):837–45.

15. Fountoulakis KN, Gonda X. Differential correlation of suicide and homicide rates according to geographical areas: A study with population-level data. Psychiatry Res. 2017;249:167–71.

16. Bando DH, Lester D. An ecological study on suicide and homicide in Brazil. Cien Saude Colet. 2014;19(4):1179–89.

17. World Health Organization. International statistical classification of diseases and related health problems: 10th revision ICD-10. Geneva: World Health Organization; 1992.

18. Fundação Nacional da Saúde. Manual de Instruções para o Preenchimento da Declaração de Óbito [Internet]. Brasília - Brazil: Ministério da Saúde; 2001. Available from: http://bvsms.saude.gov.br/bvs/publicacoes/manual_declaracao_obitos.pdf

19. Jorge MHP de M, Gotlieb SLD, Laurenti R. O sistema de informações sobre

For peer review only - http://bmjopen.bmj.com/site/about/guidelines.xhtml

mortalidade: problemas e propostas para o seu enfrentamento I - Mortes por causas naturais. Rev Bras Epidemiol. 2002;5(2):197–211.

20. Bertolote JM, Fleischmann A, De Leo D, Wasserman D. Psychiatric diagnoses and suicide: revisiting the evidence. Crisis. 2004;25(4):147–55.

21. Machado DB, Rodrigues LC, Rasella D, Barreto ML, Araya R. Conditional cash transfer programme: Impact on homicide rates and hospitalisations from violence in Brazil. PLoS One. 2018;13(12):e0208925.

22. Alves FJO, Machado DB, Barreto ML. Effect of the Brazilian cash transfer programme on suicide rates: a longitudinal analysis of the Brazilian municipalities. Soc Psychiatry Psychiatr Epidemiol. 2019;54(5):599–606.

23. Wooldridge JM. Introductory Econometrics: A Modern Approach. Econ Anal. 2003;

24. Hilbe JM. Negative binomial regression. Cambridge University Press; 2011.

25. Szwarcwald CL. Strategies for improving the monitoring of vital events in Brazil. Int J Epidemiol. 2008;37(4):738–44.

26. StataCorp. Stata Statistical Software: Release 15. College Station, TX: StataCorp LP; 2017.

27. Leenaars AA, Lester D. Suicide and homicide rates in Canada and the United States. Suicide Life-Threatening Behav. 1994;24(2):184–91.

28. Holinger PC, Klemen EH. Violent deaths in the United States, 1900–1975: Relationships between suicide, homicide and accidental deaths. Soc Sci Med. 1982;16(22):1929–38.

29. Kennedy HG, Iveson RCY, Hill O. Violence, homicide and suicide: strong correlation and wide variation across districts. Br J Psychiatry. 1999;175(5):462–6.

For peer review only

30. Hutchinson G. Variation of homicidal and suicidal behaviour within Trinidad and Tobago and the associated ecological risk factors. West Indian Med J. 2005;54(5):319–24.

31. Lee AYS, Pridmore S. Emerging correlations between measures of population well-being, suicide and homicide: a look at global and Australian data. Australas Psychiatry. 2014;22(2):112–7.

32. Ministério da Saúde. Reforma Psiquiátrica e política de Saúde Mental no Brasil: 15 anos depois de Caracas [Internet]. 2005. Available from: http://portal.saude.gov.br/portal/arquivos/pdf/relatorio_15_anos_caracas.pdf

33. Ministério da Saúde. Portaria GM/MS 3088 [Internet]. Ministério da Saúde, editor. 2011. Available from: http://bvsms.saude.gov.br/bvs/saudelegis/gm/2011/prt3088_23_12_2011_rep.htm l

34. Rodríguez Andrés A, Hempstead K. Gun control and suicide: The impact of state firearm regulations in the United States, 1995-2004. Health Policy (New York). 2011;101(1):95–103.

35. Killias M, Van Kesteren J, Rindlisbacher M. Guns, violent crime, and suicide in 21 countries. Can J Criminol. 2001;43:429.

36. Cerqueira D, De Mello J. Evaluating a national anti-firearm law and estimating the causal effect of guns on crime [Internet]. Texto para discussão; 2013. Available from: https://econpapers.repec.org/RePEc:rio:texdis:607

37. de Fátima Marinho de Souza M, Macinko J, Alencar AP, Malta DC, de Morais Neto OL. Reductions In Firearm-Related Mortality And Hospitalizations In Brazil After Gun Control. Health Aff. 2007;26(2):575–84.

For peer review only - http://bmjopen.bmj.com/site/about/guidelines.xhtml

Figure 1. Homide rate per 100,000 and age-standardised suicide rate per 100,000 in Brazil between 2008 and 2014.

For peer review only

For peer review only - http://bmjopen.bmj.com/site/about/guidelines.xhtml

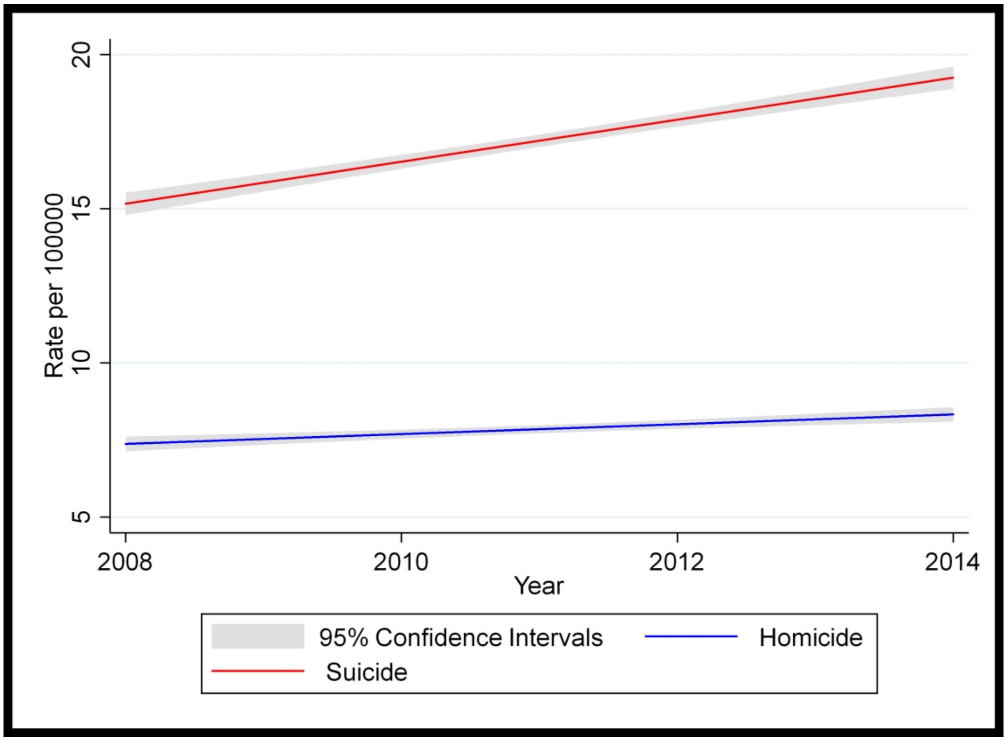

Figure 1. Homide rate per 100,000 and age-standardised suicide rate per 100,000 in Brazil between 2008 and 2014.

Supplementary Material

Machado DB, McDonald K, Castro-de-Araujo LFS, Devakumar D, Alves FJO, et al. **The association between homicide rates and suicide rates: a countrywide longitudinal analysis of 5,507 Brazilian municipalities.**

Contents

1. **Supplemental Methods**. Additional details about CAPS coverage
2. **Supplemental Figure 1.** Distribution of age-standardised suicide rates per 100,000.
3. **Supplemental Table 1.** Unadjusted and adjusted associations between age-standardized suicide and age-standardised homicide rates in all Brazilian municipalities, only municipalities with accurate vital information in Brazil, and by population size, 2008-2014.
4. **Supplemental Table 2**. Unadjusted and adjusted associations between suicide rates and homicide rates in all Brazilian municipalities for 2010 only.
5. **Supplemental References**

For peer review only

For peer review only - http://bmjopen.bmj.com/site/about/guidelines.xhtml

Supplemental Methods: Additional details about CAPS coverage

The Psychosocial Community Centers (CAPS) were developed to provide day hospital care severe mental disorders. There are five different types of CAPS, classified based on their complexity, population coverage, and funding. CAPS I are based in small towns (20,000-50,000 inhabitants), CAPS II are based in mid-sized cities (50,000 or more inhabitants), and CAPS III are large units intended to provide 24-hour services. CAPSi provide services to children and adolescents. CAPSad are specialised units to treat problems associated with drug and alcohol use. The Brazilian government estimates CAPS coverage within each municipality according to the proportion of the population served by the different types of units (1,2). Specifically, this is calculated as:
{[(number of CAPS I x 0.5) + (number of CAPS II) + (number of CAPS III x 1.5) + (number of CAPSi) + (number of CAPSad)] x 100,000} / municipal population

For peer review only

For peer review only - http://bmjopen.bmj.com/site/about/guidelines.xhtml

Supplemental Figure 1. Distribution of age-standardised suicide rates per 100,000.

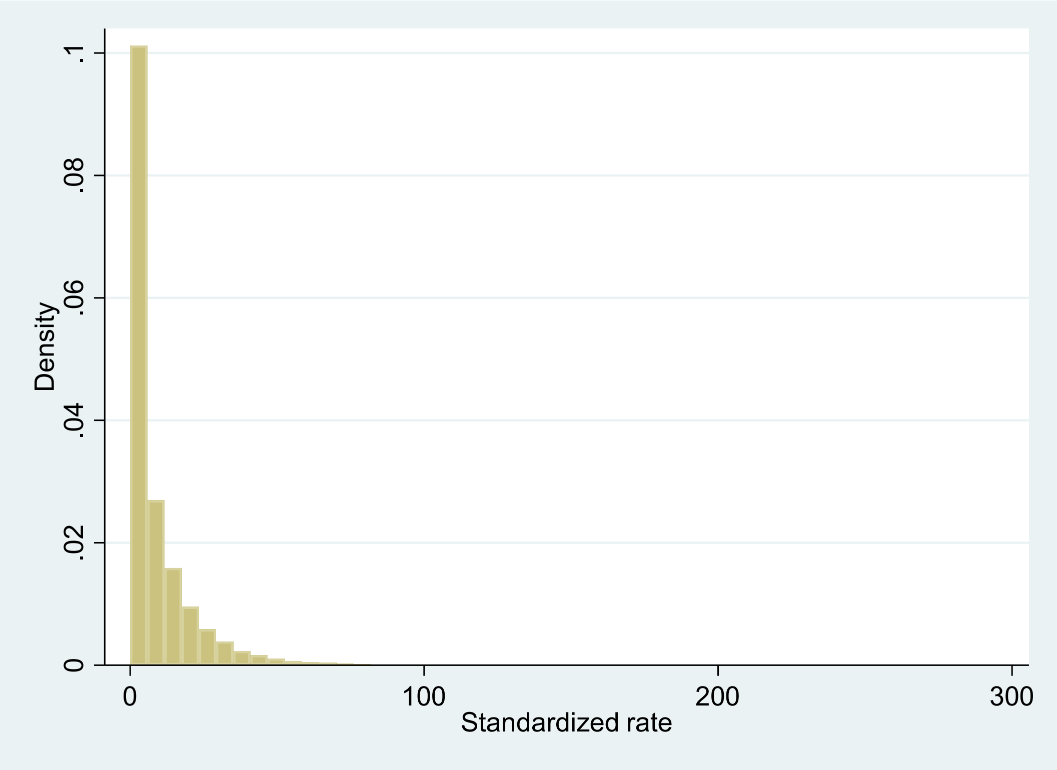

For peer review only

For peer review only - http://bmjopen.bmj.com/site/about/guidelines.xhtml

Supplemental Table 1. Unadjusted and adjusted associations between age-standardized suicide and age-standardised homicide rates in all Brazilian municipalities, only municipalities with accurate vital information in Brazil, and by population size, 2008-2014.

	**Unadjusted model**		**Adjusted model[a]**			
	**RR[b]**	**95% CI**	**RR[b]**	**95% CI**	**Number of suicides**	**Number of municipalities[c]**
All Brazilian municipalities	1.27	1.17-1.38	1.24	1.14-1.35	35,357	5,051
Municipalities with accurate vital information	1.46	1.23-1.72	1.45	1.22-1.72	9,336	1,556
Population <=10,000	1.21	1.03-1.41	1.21	1.03-1.41	14,352	2,100
Population 10,001-50,000	1.30	1.14-1.48	1.28	1.12-1.46	16,583	2,443
Population >50,000	1.33	1.15-1.55	1.32	1.13-1.54	4,259	639

Abbreviations: RR=Rate ratio; CI=Confidence interval.

[a] Models adjusted for monthly per capita income (BR$), CAPS coverage, urbanisation, percentage of people unemployed, percentage of individuals who were divorced, percentage of Pentecostal Christians, and percentage of households with one resident.

[b] Change in suicide rate per 100% increase in homicide rate.

[c] This number excludes municipalities with zero suicides recorded between 2008 and 2014.

For peer review only - http://bmjopen.bmj.com/site/about/guidelines.xhtml

Supplemental Table 2. Unadjusted and adjusted associations between suicide rates and homicide rates in all Brazilian municipalities for 2010 only.

	Unadjusted model		Adjusted model[a]			
Year	RR[b]	95% CI	RR[b]	95% CI	Number of suicides	Number of municipalities[c]
2010	0.51	0.46-0.64	0.91	0.77-1.08	9287	5,506

Abbreviations: RR=Rate ratio; CI=Confidence interval.
[a] Models adjusted for monthly per capita income (BR$), CAPS coverage, urbanisation, percentage of people unemployed, percentage of individuals who were divorced, percentage of Pentecostal Christians, and percentage of households with one resident.
[b] Change in suicide rate per 100% increase in homicide rate.
[c] This number excludes municipalities with zero suicides recorded in 2010.

For peer review only

Supplemental References

1. Ministério da Saúde. Portaria GM/MS 336 [Internet]. Ministério da Saúde, editor. 2002. Available from: http://www.saude.mg.gov.br/images/documentos/Portaria_336.pdf
2. Ministério da Saúde. Portaria GM/MS 2.669 [Internet]. Ministério da Saúde, editor. 2009. Available from: http://bvsms.saude.gov.br/bvs/saudelegis/gm/2009/prt2669_03_11_2009.html

For peer review only

STROBE Statement—checklist of items that should be included in reports of observational studies

	Item No	Recommendation	Page No
**Title and abstract**	1	(*a*) Indicate the study's design with a commonly used term in the title or the abstract<br><br>We conducted a longitudinal ecological study using annual mortality data from 5,507 Brazilian municipalities between 2008 and 2014.	2
		(*b*) Provide in the abstract an informative and balanced summary of what was done and what was found<br><br>**Objective:** To estimate the association between homicide rates and suicide rates in Brazilian municipalities over a period of 7 years.<br>**Design:** We conducted a longitudinal ecological study using annual mortality data from 5,507 Brazilian municipalities between 2008 and 2014. Multivariable negative binomial regression models were used to examine the relationship between homicide and suicide rates. Robustness of results was explored using sensitivity analyses to examine the influence of data quality, population size, age, and sex on the relationship between homicide and suicide rates.<br>**Setting:** A nationwide study of municipalities-level data.<br>**Participants:** Mortality data and corresponding population estimates for municipal populations aged 10 years and older.<br>**Primary and Secondary Outcome Measures:** Age-standardised suicide rates per 100,000.<br>**Results:** Municipal suicide rates were positively associated with municipal homicide rates; after adjusting for socioeconomic and demographic factors, a doubling of the homicide rate was associated with 22% increase in suicide rate (RR=1.22, 95% CI 1.26-1.33). A dose-response effect was observed with 4% increase in suicide rates at the third quintile, 9% at the fourth quintile and 12% at the highest quintile of homicide rates compared with the lowest quintile. The observed effect estimates were robust to sensitivity analyses.<br>**Conclusions:** Municipalities with higher homicide rates have higher suicide rates, and the relationship between homicide and suicide rates in Brazil exists independently of many sociodemographic and socioeconomic factors. The identification of a positive relationship between homicide and suicide rates suggests that population-based interventions to reduce homicide rates may also reduce suicide rates in Brazil.	2
**Introduction**			
Background/rationale	2	Explain the scientific background and rationale for the investigation being reported<br><br>Suicide is an important global public health problem, causing over 800,000 deaths per year….<br>In 2008, the World Health Organization (WHO) reported violence as a leading cause of death and disability and a "global challenge" (1)…	6-7

For peer review only

		While often studied separately, there is a long tradition of viewing homicide and suicide as related phenomena (13). Homicide and suicide can be viewed similarly as forms of violence (1). Several studies have examined the relationship between homicide and suicide rates in the population; however, evidence is mixed (see Research in Context panel). There appear to be strong variations in the magnitude and direction of association across countries (14,15). Many of the previous studies of the association have been conducted in HICs. However, the relationship between homicide and suicide rates in LMICs might be different due to different distributions and influences of risk factors in the population. There has been only one previous study of the ecological association between homicide and suicide rates in Brazil, which observed an inverse correlation in 2010 (16). However, this study used data from a single year, studied large, heterogeneous geographical areas (27 Brazilian states), and did not adjust for important demographic and socioeconomic factors.	
Objectives	3	State specific objectives, including any prespecified hypotheses<br><br>We aimed to further investigate the possible association between homicide and suicide rates in Brazil, using a dataset of all Brazilian municipalities from 2008 to 2014.	7
**Methods**			
Study design	4	Present key elements of study design early in the paper<br><br>This study used a longitudinal ecological design, using yearly data from the 5,507 Brazilian municipalities for the period 2008 to 2014.	7
Setting	5	Describe the setting, locations, and relevant dates, including periods of recruitment, exposure, follow-up, and data collection<br><br>See (4).	7
Participants	6	(*a*) *Cohort study*—Give the eligibility criteria, and the sources and methods of selection of participants. Describe methods of follow-up<br>*Case-control study*—Give the eligibility criteria, and the sources and methods of case ascertainment and control selection. Give the rationale for the choice of cases and controls<br>*Cross-sectional study*—Give the eligibility criteria, and the sources and methods of selection of participants<br><br>Ecological study design:<br><br>All data used in this study are publicly available online (http://www2.datasus.gov.br). To estimate suicide and homicide rates, cause-specific mortality data were collected from the Brazilian Ministry of Health's Mortality Information System. All deaths are recorded in this system using the International Classification of Diseases, 10th revision (17). Socioeconomic, demographic variables (monthly per capita income in Brazilian Real (BR$); unemployment rate; percentage of individuals who were divorced; percentage of individuals who declared being Pentecostal; percentage of households with only one resident; and urbanisation), and	8-9

For peer review only - http://bmjopen.bmj.com/site/about/guidelines.xhtml

For peer review only

		municipality population estimates were obtained from the Brazilian Institute of Geography and Statistics (IBGE). Data from the main mental health services in Brazil, the Psychosocial Care Centre (CAPS) coverage was obtained from the mental health coordination database, held by the Ministry of Health (DATASUS). …<br><br>Suicide is defined as a death resulting from intentional self-harm according to the International Classification of Diseases, 10th revision (ICD-10) codes X60 to X84 (17). …<br><br>Suicides among individuals under 10 years-old were excluded because suicide is a very rare event before 10 years; there were 33 victims of suicide under 10 years in Brazil in the period 2008-2014 (http://www2.datasus.gov.br). …<br><br>Homicide was defined as a death resulting from injuries inflicted by another person with the intent to injure or kill using any means according to the ICD-10 codes X85 to Y09 (17).	
		(*b*) *Cohort study*—For matched studies, give matching criteria and number of exposed and unexposed<br>*Case-control study*—For matched studies, give matching criteria and the number of controls per case	
Variables	7	Clearly define all outcomes, exposures, predictors, potential confounders, and effect modifiers. Give diagnostic criteria, if applicable<br><br>See (6). Also:<br><br>Municipality-level covariates for suicide considered in the model included: the monthly per capita income in Brazilian Real (BR$); unemployment rate, measured by the percentage of the population aged 16 years or over with no official job; percentage of individuals who were divorced; percentage of individuals who declared being Pentecostal; percentage of households with only one resident; and urbanisation, measured as the percentage of individuals living in urban areas for each municipality. These variables were selected based on evidence in the literature that they may be associated with suicide (3,20–22).	7-10
Data sources/ measurement	8*	For each variable of interest, give sources of data and details of methods of assessment (measurement). Describe comparability of assessment methods if there is more than one group<br><br>See (6) and (7).	7-10
Bias	9	Describe any efforts to address potential sources of bias<br><br>To examine the robustness of our primary findings, we fitted several additional negative binomial regression models: one including only municipalities considered to have very accurate vital information according to a previous study (25), three by total municipal population (10,000	12

For peer review only - http://bmjopen.bmj.com/site/about/guidelines.xhtml

		inhabitants; 10,001-50,000; and more than 50,000 inhabitants), one by municipal homicide rates quintiles, and six further stratified by sex and age groups (10-19 years; 20-59 years; 60 and older). Finally, we tested the association between homicide rates and age-standardised suicide rates for 2010 only to enable a comparison with the results of a previous study of Brazilian states (16). For each analysis, we fitted both unadjusted and adjusted models. Statistical analyses were performed using Stata 15 software (26).	
Study size	10	Explain how the study size was arrived at<br><br>Not applicable; the study was based on all municipalities in Brazil.	N/A

For peer review only

Quantitative variables	11	Explain how quantitative variables were handled in the analyses. If applicable, describe which groupings were chosen and why<br><br>See (7). Also Supplementary Methods and:<br>The main estimated regression model was: $\log(Yit) = \alpha i + \beta 1HOMICIDEit + \beta nXnit + \gamma t + uit$, where t =year and i =municipality, Yit is the standardized mortality rate (per 100,000 inhabitants) due to suicide, αi is the fixed effect for municipality i capturing unobserved time-invariant factors causing between-municipality variability in suicide, HOMICIDEit was the rate of homicide for the municipality i in the year t, Xnit is the value taken by a covariate vector containing the n socioeconomic and demographic determinants included in the model, for municipality i in year t, γt is a time-specific effect, capturing variability in suicide rates over time, uit is an error term, assumed to follow a normal distribution. To produce rate ratio (RR) estimates that were more readily interpretable, the homicide rate was divided by 100 (i.e. homicide rate per 1,000).<br><br>The age-standardised suicide rate, although not a count, still appeared to follow a binomial distribution (Supplemental Figure 1). We compared a negative binomial model to a Poisson regression model using an Akaike's Information Criterion and the Bayesian Information Criterion to establish the model that best fitted the data (24). We found evidence that the age-standardised suicide rates varied between municipalities more than expected under a Poisson distribution, therefore, the negative binomial model was used to allowed for overdispersion. The Hausman test was used to compare a negative binomial regression with fixed effects to a model with a random effect for each municipality. The test showed no evidence that the random effects model provided a better fit to the data (p=0.05), therefore, our model included only fixed effects.<br><br>Population standardization was used to allow comparability across municipalities with distinct age structures. We also tested the association using models based on both age-standard homicide rates and age-standardised suicide rates, and the results were similar (Supplemental Table 1).	7-10, Supplementary Materials, 10-11
Statistical methods	12	(*a*) Describe all statistical methods, including those used to control for confounding<br><br>See (7) and (11).. Also:<br>For each analysis, we fitted both unadjusted and adjusted models.	7-10, 11
		(*b*) Describe any methods used to examine subgroups and interactions<br><br>See (11).	11
		(*c*) Explain how missing data were addressed<br><br>Not applicable.	N/A
		(*d*) *Cohort study*—If applicable, explain how loss to follow-up was addressed	

For peer review only

Case-control study—If applicable, explain how matching of cases and controls was addressed<br>*Cross-sectional study*—If applicable, describe analytical methods taking account of sampling strategy	
(*e*) Describe any sensitivity analyses<br><br>See (9).	12

Continued on next page

For peer review only

**Results**			
Participants	13*	(a) Report numbers of individuals at each stage of study—eg numbers potentially eligible, examined for eligibility, confirmed eligible, included in the study, completing follow-up, and analysed<br><br>Not applicable. The number of municipalities (n=5,506 are reported in the title, abstract and methods). Numbers of events (suicides) and municipalities in each analysis are reported in the tables/table legends.	N/A
		(b) Give reasons for non-participation at each stage<br><br>Not applicable.	N/A
		(c) Consider use of a flow diagram<br><br>Not applicable.	N/A
Descriptive data	14*	(a) Give characteristics of study participants (eg demographic, clinical, social) and information on exposures and potential confounders<br><br>See Table 1.	13
		(b) Indicate number of participants with missing data for each variable of interest<br><br>Not applicable.	N/A
		(c) *Cohort study*—Summarise follow-up time (eg, average and total amount)<br><br>Not applicable.	N/A
Outcome data	15*	*Cohort study*—Report numbers of outcome events or summary measures over time<br><br>See Tables 2-4.	14, 16-17
		Case-control study—Report numbers in each exposure category, or summary measures of exposure	
		Cross-sectional study—Report numbers of outcome events or summary measures	
Main results	16	(*a*) Give unadjusted estimates and, if applicable, confounder-adjusted estimates and their precision (eg, 95% confidence interval). Make clear which confounders were adjusted for and why they were included<br><br>See Tables 2-4. Columns of unadjusted and adjusted estimates are provided. Confounders are listed in the table legends.	14, 16-17
		(*b*) Report category boundaries when continuous variables were categorized	
		(*c*) If relevant, consider translating estimates of relative risk into absolute risk for a meaningful time period	
Other analyses	17	Report other analyses done—eg analyses of subgroups and interactions, and sensitivity analyses<br><br>Analysing by quintile of homicide rate showed that the higher the homicide rate, the stronger the association, with 1% suicide rate increase at the second quintile,	15-18

For peer review only - http://bmjopen.bmj.com/site/about/guidelines.xhtml

		4% increase at the third quintile, 9% increase at the fourth quintile, and 12% at the highest quintile compared with those in the lowest quintile (Table 3). The analysis stratified by sex and age groups showed a positive association between homicide rates and suicide among men from 20 to 59 years old (Table 4).<br><br>Table 3<br><br>Table 4<br><br>We repeated the analysis based on data from 2010 only. The unadjusted analysis showed a significant inverse relationship between homicide rates and suicide rates in 2010 (RR=0.51, 95% CI: 0.746-0.64). However, after adjusting for confounders the estimate of association became closer to the null value (RR=1.0) and was longer significant (RR=0.91, 95% CI: 0.77-1.08; Supplemental Table 2).	
**Discussion**			
Key results	18	Summarise key results with reference to study objectives<br><br>We assessed the relationship between homicide and suicide rates in Brazilian municipalities over a 7-year period. We found that municipalities with higher homicide rates also had higher age-standardised suicide rates in both unadjusted and adjusted models. Our results appeared to be robust based on several sensitivity analyses. Similar results were observed for models stratified by population size, and for models including only municipalities considered to have accurate data (25). The association between homicide and suicide rates seemed to follow a dose-response pattern, such that areas with higher homicide rates showed stronger relationships with increased suicide rates, reaching 12% increase in the highest quintile of homicide rates compared with the lowest.	18
Limitations	19	Discuss limitations of the study, taking into account sources of potential bias or imprecision. Discuss both direction and magnitude of any potential bias<br><br>This study used an ecological design, and therefore conclusions are applicable to populations rather than individuals. An ecological design was used due to data availability, and only the use of aggregate data allows the study of the total Brazilian population longitudinally. Furthermore, given our interest in homicide rates as an indicator of contextual violence, aggregate data for homicide were required.<br><br>Another limitation could be that large municipalities and small municipalities have equal weight in the analysis. For this reason, we have also provided estimates after stratification by municipal population size.<br><br>Potential bias resulting from misclassification of suicides and homicides is another possible limitation. Homicide and suicide deaths may have a risk of misclassification as events of undetermined intent or unspecified accidents. Suicide may be underreported due to stigmatisation and social taboo, while homicides could be under-reported as they may indicate unrest or instability. It is unclear whether the frequency of misclassification differs between homicides and suicides. If misclassification of homicides and suicides occurred equally	18-19

For peer review only - http://bmjopen.bmj.com/site/about/guidelines.xhtml

		frequently, then the observed estimates of association between homicide and suicide rates are conservative. In fact, the observed estimate of association was slightly stronger when only municipalities judged to have high-quality vital information were included. Nevertheless, misclassification is not expected to be a major concern because all deaths due to reported external causes, including homicides and suicides, are determined based on autopsy (19).	
Interpretation	20	Give a cautious overall interpretation of results considering objectives, limitations, multiplicity of analyses, results from similar studies, and other relevant evidence<br><br>The results of this study are in contrast with a previous cross-sectional ecological study of the association between homicide and suicide rates in Brazil, which observed a significant inverse correlation between the two forms of death. …<br><br>In the broader literature, existing studies of the relationship between homicide and suicide rates provide mixed evidence. …<br><br>We observed that the relationship between homicide and suicide rates persisted even after adjusting for several important factors including per capita income, CAPS coverage, urbanisation, unemployment, single-person households, divorce, and religious affiliation. Therefore, we have evidence that, in Brazil, the relationship exists, even after accounting for these factors.	20-22
Generalisability	21	Discuss the generalisability (external validity) of the study results<br><br>We observed that the relationship between homicide and suicide rates persisted even after adjusting for several important factors including per capita income, CAPS coverage, urbanisation, unemployment, single-person households, divorce, and religious affiliation. Therefore, we have evidence that, in Brazil, the relationship exists, even after accounting for these factors.	22
**Other information**			
Funding	22	Give the source of funding and the role of the funders for the present study and, if applicable, for the original study on which the present article is based<br><br>See Funding.	24

*Give information separately for cases and controls in case-control studies and, if applicable, for exposed and unexposed groups in cohort and cross-sectional studies.

Note: An Explanation and Elaboration article discusses each checklist item and gives methodological background and published examples of transparent reporting. The STROBE checklist is best used in conjunction with this article (freely available on the Web sites of PLoS Medicine at http://www.plosmedicine.org/, Annals of Internal Medicine at http://www.annals.org/, and Epidemiology at http://www.epidem.com/). Information on the STROBE Initiative is available at www.strobe-statement.org.

For peer review only - http://bmjopen.bmj.com/site/about/guidelines.xhtml

The association between homicide rates and suicide rates: a countrywide longitudinal analysis of 5,507 Brazilian municipalities

Journal:	*BMJ Open*
Manuscript ID	bmjopen-2020-040069.R1
Article Type:	Original research
Date Submitted by the Author:	16-Sep-2020

Complete List of Authors:	Machado, Daiane Borges; Center of Data and Knowledge Integration for Health<br>McDonald, Keltie; University College London, Division of Psychiatry<br>Araújo , Luis ; Center of Data and Knowledge Integration for Health<br>Devakumar, Delan; University College London Research Department of Epidemiology and Public Health,<br>Alves, Flávia; Center of Data and Knowledge Integration for Health<br>Kiss, Lígia; University College London, Institute for Global Health<br>Lewis, Glyn; University College London, Division of Psychiatry<br>Barreto, Mauricio; Federal University of Bahia, Instituto de Saúde Coletiva

**Primary Subject Heading**:	Epidemiology
Secondary Subject Heading:	Mental health, Public health
Keywords:	EPIDEMIOLOGY, PUBLIC HEALTH, PSYCHIATRY, MENTAL HEALTH, Suicide & self-harm < PSYCHIATRY

I, the Submitting Author has the right to grant and does grant on behalf of all authors of the Work (as defined in the below author licence), an exclusive licence and/or a non-exclusive licence for contributions from authors who are: i) UK Crown employees; ii) where BMJ has agreed a CC-BY licence shall apply, and/or iii) in accordance with the terms applicable for US Federal Government officers or employees acting as part of their official duties; on a worldwide, perpetual, irrevocable, royalty-free basis to BMJ Publishing Group Ltd ("BMJ") its licensees and where the relevant Journal is co-owned by BMJ to the co-owners of the Journal, to publish the Work in this journal and any other BMJ products and to exploit all rights, as set out in our licence.

The Submitting Author accepts and understands that any supply made under these terms is made by BMJ to the Submitting Author unless you are acting as an employee on behalf of your employer or a postgraduate student of an affiliated institution which is paying any applicable article publishing charge ("APC") for Open Access articles. Where the Submitting Author wishes to make the Work available on an Open Access basis (and intends to pay the relevant APC), the terms of reuse of such Open Access shall be governed by a Creative Commons licence – details of these licences and which Creative Commons licence will apply to this Work are set out in our licence referred to above.

Other than as permitted in any relevant BMJ Author's Self Archiving Policies, I confirm this Work has not been accepted for publication elsewhere, is not being considered for publication elsewhere and does not duplicate material already published. I confirm all authors consent to publication of this Work and authorise the granting of this licence.

For peer review only

The association between homicide rates and suicide rates: a countrywide longitudinal analysis of 5,507 Brazilian municipalities

Daiane Borges Machado[1,2]*, Keltie McDonald[3]*, Luís F S Castro-de-Araujo[1,4], Delanjathan Devakumar[5], Flávia Jôse Oliveira Alves[1], Lígia Kiss[5], Glyn Lewis[3], Maurício L. Barreto[1,6].

*Denotes equal contribution as first author

Corresponding author: Keltie McDonald; k.mcdonald@ucl.ac.uk; Division of Psychiatry, University College London, 6th Floor Maple House, 149 Tottenham Court Road, London, W1T 7NF, UK.

[1] Center of Data and Knowledge Integration for Health (CIDACS), Salvador, Brazil.

[2] Centre for Global Mental Health, London School of Hygiene & Tropical Medicine (LSHTM).

[3] Division of Psychiatry, University College London, London, United Kingdom.

[4] The University of Melbourne, Department of Psychiatry, Austin Health, Heidelberg, Victoria, Australia.

[5] Institute for Global Health, University College London, London, United Kingdom.

[6] Institute of Collective Health, Federal University of Bahia, Salvador, Brazil.

Keywords: homicide, suicide, ecological study, longitudinal study, epidemiology, public health, mental health

Word count: 3,883

For peer review only - http://bmjopen.bmj.com/site/about/guidelines.xhtml

ABSTRACT

Objective: To estimate the association between homicide and suicide rates in Brazilian municipalities over a period of 7 years.

Design: We conducted a longitudinal ecological study using annual mortality data from 5,507 Brazilian municipalities between 2008 and 2014. Multivariable negative binomial regression models were used to examine the relationship between homicide and suicide rates. Robustness of results was explored using sensitivity analyses to examine the influence of data quality, population size, age, and sex on the relationship between homicide and suicide rates.

Setting: A nationwide study of municipalities-level data.

Participants: Mortality data and corresponding population estimates for municipal populations aged 10 years and older.

Primary and Secondary Outcome Measures: Age-standardised suicide rates per 100,000.

Results: Municipal suicide rates were positively associated with municipal homicide rates; after adjusting for socioeconomic and demographic factors, a doubling of the homicide rate was associated with 22% increase in suicide rate (RR=1.22, 95% CI 1.26-1.33). A dose-response effect was observed with 4% increase in suicide rates at the third quintile, 9% at the fourth quintile and 12% at the highest quintile of homicide rates compared with the lowest quintile. The observed effect estimates were robust to sensitivity analyses.

Conclusions: Municipalities with higher homicide rates have higher suicide rates and the relationship between homicide and suicide rates in Brazil exists independently of many sociodemographic and socioeconomic factors. Our results are in line with the hypothesis that changes in homicide rates lead to changes in suicide rates, although a

For peer review only - http://bmjopen.bmj.com/site/about/guidelines.xhtml

causal association cannot be established from this study. Suicide and homicide rates have increased in Brazil despite increased community mental health support and incarceration, respectively, therefore new avenues for intervention are needed. The identification of a positive relationship between homicide and suicide rates suggests that population-based interventions to reduce homicide rates may also reduce suicide rates in Brazil.

For peer review only

For peer review only - http://bmjopen.bmj.com/site/about/guidelines.xhtml

STRENGTHS AND LIMITATIONS OF THIS STUDY

- In this longitudinal ecological study, we examined the association between homicide and suicide rates using data from 5,507 municipalities in Brazil from 2008 to 2014, including several sensitivity analyses to address limitations of previous examinations of this topic.
- Homicide and suicide are important public health concerns in Brazil and globally, and our findings provide clear evidence for their relationship, and suggest potential avenues for public health and policy interventions to reduce deaths from these causes.
- Our study used an ecological design, and therefore, results are generalisable to the municipal population-level and not to individuals.

For peer review only

For peer review only - http://bmjopen.bmj.com/site/about/guidelines.xhtml

INTRODUCTION

Suicide is an important global public health problem, causing over 800,000 deaths per year. Amongst 15-29 year-olds, suicide is the second leading cause of mortality worldwide (1). One of the strongest and most consistently identified risk factors for suicide is psychiatric disorder (2). In Brazil in 2012, the national rate of suicide was 6.2 per 100,000 inhabitants, and was higher in males (10.0 per 100,000) than females (2.7 per 100,000) (3). The rate of suicide has increased in nearly all age groups for males and females between 2000 and 2016 (4).

In 2008, the World Health Organization (WHO) reported violence as a leading cause of death and disability and a "global challenge" (1). Violence is not exclusively a criminal justice issue; it is also a health and human rights issue. Over 90% of deaths caused by violence occur in low and middle income countries (LMICs), however, most studies into violent deaths have been carried out in high-income countries (HICs) (5). In Brazil, the homicide rate has increased over the last three decades (6). In 2016, interpersonal violence was the second leading cause of age-standardised years of life lost in Brazil (7).

Brazil is the largest country in Latin America with 207 million inhabitants. Despite, being the 9th richest country in the world in terms of nominal Gross Domestic Product, Brazil has one of the highest levels of income inequality in the world (8,9). Homicide in Brazil has been related to socioeconomic inequalities (6,10,11), poverty (12), race, gender and age, with men, younger people, and those identifying as black at higher risk

For peer review only

(6,10), while suicide has also been associated with income inequality, sex, and age and low levels of education (3,4).

While often studied separately, there is a long tradition of viewing homicide and suicide as related phenomena (13). Homicide and suicide can be viewed similarly as forms of violence (1). Several studies have examined the relationship between homicide and suicide rates in the population; however, evidence is mixed. There appear to be strong variations in the magnitude and direction of association across countries and regions (14,15). Many of the previous studies of the association have been conducted in HICs. However, the relationship between homicide and suicide rates in LMICs might be different due to different distributions and influences of risk factors in the population. There has been only one previous study of the ecological association between homicide and suicide rates in Brazil, which observed an inverse correlation in 2010 (16). However, this study used data from a single year, studied large, heterogeneous geographical areas (27 Brazilian states), and did not adjust for important demographic and socioeconomic factors. We aimed to further investigate the possible association between homicide and suicide rates in Brazil, using a dataset of all Brazilian municipalities from 2008 to 2014.

METHODS

Study Design

This study used a longitudinal ecological design, using yearly data from the 5,507 Brazilian municipalities for the period 2008 to 2014.

For peer review only - http://bmjopen.bmj.com/site/about/guidelines.xhtml

Data Sources

All data used in this study are publicly available online (http://www2.datasus.gov.br). To estimate suicide and homicide rates, cause-specific mortality data were collected from the Brazilian Ministry of Health's Mortality Information System. All deaths are recorded in this system using the International Classification of Diseases, 10th revision (17). Socioeconomic, demographic variables (monthly per capita income in Brazilian Real (BR$); unemployment rate; percentage of individuals who were divorced; percentage of individuals who declared being Pentecostal; percentage of households with only one resident; and urbanisation), and municipality population estimates were obtained from the Brazilian Institute of Geography and Statistics (IBGE). Data from the main mental health services in Brazil, the Psychosocial Care Centre (CAPS) coverage was obtained from the mental health coordination database, held by the Ministry of Health (DATASUS).

Definition of variables

Outcome

Suicide is defined as a death resulting from intentional self-harm according to the International Classification of Diseases, 10th revision (ICD-10) codes X60 to X84 (17). In Brazil, all deaths due to external causes (suicide, homicide, and accidents) are forwarded to the Medical Legal Institute (IML; artigo 2º da Resolução CFM nº. 1.779/2005) where the death certificates are printed and signed by an examining doctor (18), which decreases the chances for misclassification. Diagnoses are based on an autopsy (using the ICD-10 classification), to investigate the circumstances in which the death occurred, personal history of the victim, and suicide risk factors (19).

For peer review only - http://bmjopen.bmj.com/site/about/guidelines.xhtml

The suicide rate was calculated at the municipality level and as it is influenced by age, we standardized by each 5-year age range, from 10 years or older. We used direct standardisation using the WHO population distribution as the standard population. Suicides among individuals under 10 years-old were excluded because suicide is a very rare event before 10 years; there were 33 victims of suicide under 10 years in Brazil in the period 2008-2014 (http://www2.datasus.gov.br). Overall and sex-specific age-standardised suicide rates were calculated for each municipality and year of analysis, expressed as a rate per 100,000 inhabitants.

Exposure

Homicide was defined as a death resulting from injuries inflicted by another person with the intent to injure or kill using any means according to the ICD-10 codes X85 to Y09 (17). Homicide rates were also calculated at the municipality level. Homicide rates were not standardized by age because it was used as proxy for violence, and therefore, total violence irrespective of age distribution was of interest. Overall and sex-specific homicide rates were calculated for each municipality and year of analysis, expressed as a rate per 100,000 inhabitants.

Confounders

Municipality-level covariates for suicide considered in the model included: the monthly per capita income in Brazilian Real (BR$); unemployment rate, measured by the percentage of the population aged 16 years or over with no official job; percentage of individuals who were divorced; percentage of individuals who declared being Pentecostal; percentage of households with only one resident; and urbanisation,

For peer review only - http://bmjopen.bmj.com/site/about/guidelines.xhtml

measured as the percentage of individuals living in urban areas for each municipality. These variables were selected based on evidence in the literature that they may be associated with suicide (3,20–22).

The percentage of individuals who declared being Pentecostal was chosen as a confounder, as in previous studies (3,22), because it is likely to be the best proxy for measuring religious engagement in Brazil; Brazilians who identify themselves as Pentecostal tend to actively attend the church and its activities while those who identify themselves as Catholic seem to do so only referring to their family background, even if they do not participate in any religious activity (23).

We also included the Psychosocial Care Centre (CAPS) coverage, defined as the proportion of people who could potentially be seen in the centres, because availability of mental health care may be associated with suicide rates. CAPS coverage was calculated for each municipality based on the different types of CAPS available and the populations that they are expected to serve (Supplemental Methods).

Statistical Analysis

We used negative binomial regression analysis to evaluate the association between age-standardized suicide rates and rates of homicide. Year of study (a time-specific fixed effect) was introduced into the models to control for the national-level policy changes or secular trends that may have affected all municipalities (24).

The main estimated regression model was: $\log(Y_{it}) = \alpha_i + \beta_1 HOMICIDE_{it} + \beta_n X_{nit} + \gamma_t + u_{it}$, where t =year and i =municipality, Y_{it} is the standardized mortality rate (per 100,000

For peer review only - http://bmjopen.bmj.com/site/about/guidelines.xhtml

inhabitants) due to suicide, α_i is the fixed effect for municipality *i* capturing unobserved time-invariant factors causing between-municipality variability in suicide, $HOMICIDE_{it}$ was the rate of homicide for the municipality *i* in the year *t*, X_{nit} is the value taken by a covariate vector containing the *n* socioeconomic and demographic determinants included in the model, for municipality *i* in year *t*, γ_t is a time-specific effect, capturing variability in suicide rates over time, u_{it} is an error term, assumed to follow a normal distribution. To produce rate ratio (RR) estimates that were more readily interpretable, the homicide rate was divided by 100 (i.e. homicide rate per 1,000).

The age-standardised suicide rate, although not a count, still appeared to follow a binomial distribution (Supplemental Figure 1). We compared a negative binomial model to a Poisson regression model using an Akaike's Information Criterion and the Bayesian Information Criterion to establish the model that best fitted the data (25). We found evidence that the age-standardised suicide rates varied between municipalities more than expected under a Poisson distribution, therefore, the negative binomial model was used to allowed for overdispersion. The Hausman test was used to compare a negative binomial regression with fixed effects to a model with a random effect for each municipality. The test showed no evidence that the random effects model provided a better fit to the data (p=0.05), therefore, our model included only fixed effects.

Population standardization was used to allow comparability across municipalities with distinct age structures. We also tested the association using models based on both age-standardised homicide rates and age-standardised suicide rates, and the results were similar (Supplemental Table 1).

For peer review only - http://bmjopen.bmj.com/site/about/guidelines.xhtml

Primary Analysis

To test the association between homicide and age-standardised suicide rate we constructed a negative binomial regression model including data from all Brazilian municipalities. We produced both unadjusted models and models adjusting for confounders.

Secondary Analyses

To examine the robustness of our primary findings, we fitted several additional negative binomial regression models: one including only municipalities considered to have very accurate vital information according to a previous study (26), three by total municipal population (10,000 inhabitants; 10,001-50,000; and more than 50,000 inhabitants), one by municipal homicide rates quintiles, and six further stratified by sex and age groups (10-19 years; 20-59 years; 60 and older). Finally, we tested the association between homicide rates and age-standardised suicide rates for 2010 only to enable a comparison with the results of a previous study of Brazilian states (16). For each analysis, we fitted both unadjusted and adjusted models. Statistical analyses were performed using Stata 15 software (27).

Patient and Public Involvement

There was no patient and public involvement in this study.

RESULTS

The age-standardised suicide rate increased in the Brazilian municipalities from 7.6/100,000 in 2008 to 8.2/100,000 inhabitants in 2014. Homicide rates also increased

For peer review only - http://bmjopen.bmj.com/site/about/guidelines.xhtml

over this period, from 15.2/100,000 to 19.6/100,000 inhabitants. Unemployment decreased between 2008 and 2014, while all other covariates increased (**Table 1**).

For peer review only

Table 1. Mean values and standard deviations of suicide and homicide rates and selected covariates for the Brazilian municipalities (n=5,507) in 2008 and 2014.

	2008		2014		
**Outcome**	**Mean**	**SD**	**Mean**	**SD**	**Percentage Change**[c]
Suicide rate[a,b]	7.58	12.79	8.18	13.25	7.97
Homicide rate[b]	15.21	18.39	19.65	21.84	29.16
CAPS coverage per municipal population	0.26	0.73	0.45	0.97	72.13
Monthly per capita income (BR$)	453.30	227.58	540.53	265.35	19.24
Unemployed people (%)	7.15	3.72	4.85	4.22	-32.16
Individuals who were divorced (%)	3.37	1.75	4.13	2.10	22.73
Pentecostal Christians (%)	10.43	6.17	12.10	7.15	15.96
Households with one resident (%)	11.02	2.88	12.82	3.33	16.27
Urbanisation (%)	63.04	22.03	66.26	21.80	5.10

Abbreviations: SD=standard deviation.
[a] Age-standardised.
[b] Rate per 100,000.
[c] Percentage change from 2008.

We found a positive association between homicide rates and age-standardised suicide rates in the unadjusted model and after controlling for confounders (**Table 2**). In the model based on all Brazilian municipalities, a doubling of the homicide rate was associated with 22% increase in the suicide rate (RR=1.22, 95% CI: 1.13-1.33). We found similar results in the models stratified by municipal population sizes. Sensitivity analyses, based on a subset of municipalities considered to have accurate data, yield similar results but with a stronger association (RR=1.44, 95% CI: 1.22-1.70).

For peer review only - http://bmjopen.bmj.com/site/about/guidelines.xhtml

Table 2. Unadjusted and adjusted associations between suicide rates and homicide rates in all the Brazilian municipalities, only municipalities with accurate vital information in Brazil, and by population size, 2008-2014.

	**Unadjusted model**		**Adjusted model[a]**			
	**RR[b]**	**95% CI**	**RR[b]**	**95% CI**	**Number of suicides**	**Number of municipalities[c]**
All Brazilian municipalities	1.25	1.16 to 1.36	1.22	1.13 to 1.33	35,357	5,051
Municipalities with accurate vital information	1.45	1.23 to 1.70	1.44	1.22 to 1.70	9,336	1,556
Population ≤10,000	1.19	1.01 to 1.39	1.18	1.01 to 1.39	14,352	2,100
Population 10,001-50,000	1.29	1.13 to 1.47	1.27	1.11 to 1.45	16,583	2,443
Population >50,000	1.29	1.12 to 1.49	1.28	1.10 to 1.48	4,259	639

Abbreviations: CI=confidence interval; RR=rate ratio.
[a] Model adjusted for monthly per capita income (BR$), CAPS coverage, urbanisation, percentage of people unemployed, percentage of individuals who were divorced, percentage of Pentecostal Christians, and percentage of households with one resident.
[b] Change in suicide rate per 100% increase in homicide rate.
[c] This number excludes municipalities with zero suicides recorded between 2008 and 2014.

For peer review only

For peer review only - http://bmjopen.bmj.com/site/about/guidelines.xhtml

Analysing by quintile of homicide rate showed that the higher the homicide rate, the stronger the association, with 1% suicide rate increase at the second quintile, 4% increase at the third quintile, 9% increase at the fourth quintile, and 12% at the highest quintile compared with those in the lowest quintile (**Table 3**). The analysis stratified by sex and age groups showed a positive association between homicide rates and suicide among men from 20 to 59 years old (**Table 4**).

For peer review only

For peer review only - http://bmjopen.bmj.com/site/about/guidelines.xhtml

Table 3. Unadjusted and adjusted associations between suicide rates and homicide rates in the Brazilian municipalities by quintiles of homicide rates, 2008–2014[a]

	Unadjusted model		Adjusted model[b]	
**Homicide rate**	**RR[c]**	**95% CI**	**RR[c]**	**95% CI**
1st quintile (reference)	1		1	
2nd quintile	1.01	0.96 to 1.06	1.01	0.96 to 1.06
3rd quintile	1.04	0.99 to 1.09	1.04	0.99 to 1.09
4th quintile	1.10	1.04 to 1.15	1.09	1.04 to 1. .14
5th quintile	1.13	1.07 to 1.19	1.12	1.06 to 1.18

Abbreviations: CI=confidence interval; RR=rate ratio.
[a] Based on 35,357 suicides from 5,051 municipalities. Municipalities with zero suicides recorded between 2008 and 2014 were excluded.
[b] Model adjusted for monthly per capita income (BR$), CAPS coverage, urbanisation, percentage of unemployed people, percentage of individuals who were divorced, percentage of Pentecostal Christians, and percentage of households with one resident.
[c] Change in suicide rate per 100% increase in homicide rate.

For peer review only - http://bmjopen.bmj.com/site/about/guidelines.xhtml

Table 4. Unadjusted and adjusted associations between suicide rates and homicide rates in the Brazilian municipalities, by sex and age groups, 2008–2014.

		Unadjusted model		Adjusted model[a]			
**Age range**	**Sex**	**RR[b]**	**95% CI**	**RR[b]**	**95% CI**	**Number of suicides**	**Number of municipalities[c]**
10-19	Men	1.24	0.91-1.71	1.20	0.87-1.66	10,626	1,518
	Women	1.53	0.96-2.42	1.53	0.96-2.43	6,167	881
20-59	Men	1.29	1.17-1.43	1.24	1.12-1.38	32,382	4,626
	Women	1.23	1.00-1.51	1.20	0.98-1.47	19,187	2,741
60 and older	Men	1.12	0.90-1.39	1.05	0.84-1.32	19,096	2,728
	Women	1.39	0.89-2.18	1.28	0.80-2.01	7,455	1,065

Abbreviations: CI=confidence interval; RR=rate ratio.
[a] Model adjusted for monthly per capita income (BR$), CAPS coverage, urbanisation, percentage of unemployed people, percentage of individuals who were divorced, percentage of Pentecostal Christians, and percentage of households with one resident.
[b] Change in suicide rate per 100% increase in homicide rate.
[c] This number excludes municipalities with zero suicides recorded between 2008 and 2014.

review only

We repeated the analysis based on data from 2010 only. The unadjusted analysis showed a significant inverse relationship between homicide rates and suicide rates in 2010 (RR=0.51, 95% CI: 0.746-0.64). However, after adjusting for confounders the estimate of association became closer to the null value (RR=1.0) and was no longer significant (RR=0.91, 95% CI: 0.77-1.08; Supplemental Table 2).

DISCUSSION

Main Findings

We assessed the relationship between homicide and suicide rates in Brazilian municipalities over a 7-year period. We found that municipalities with higher homicide rates also had higher age-standardised suicide rates in both unadjusted and adjusted models. Our results appeared to be robust based on several sensitivity analyses. Similar results were observed for models stratified by population size, and for models including only municipalities considered to have accurate data (26). The association between homicide and suicide rates seemed to follow a dose-response pattern, such that areas with higher homicide rates showed stronger relationships with increased suicide rates, reaching 12% increase in the highest quintile of homicide rates compared with the lowest.

Strengths and Limitations

This study examined the relationship between homicide and suicide rates in detail. Our data covered all 5,507 municipalities in Brazil and are, therefore, representative of the country. The use of fine-grained geographical areas likely resulted in less within-area heterogeneity and provided improved opportunity to control for confounding factors.

For peer review only

The estimates of association observed in this study appear to be robust, with minimal changes in magnitude between models accounting for different potential sources of bias.

Some limitations of this study are acknowledged. This study used an ecological design, and therefore conclusions are applicable to populations rather than individuals. An ecological design was used due to data availability, and only the use of aggregate data allows the study of the total Brazilian population longitudinally. Furthermore, given our interest in homicide rates as an indicator of contextual violence, aggregate data for homicide were required.

Another limitation could be that large municipalities and small municipalities have equal weight in the analysis. For this reason, we have also provided estimates after stratification by municipal population size.

Potential bias resulting from misclassification of suicides and homicides is another possible limitation. Homicide and suicide deaths may have a risk of misclassification as events of undetermined intent or unspecified accidents. Suicide may be underreported due to stigmatisation and social taboo, while homicides could be under-reported as they may indicate unrest or instability. It is unclear whether the frequency of misclassification differs between homicides and suicides. If misclassification of homicides and suicides occurred equally frequently, then the observed estimates of association between homicide and suicide rates are conservative. In fact, the observed estimate of association was slightly stronger when only municipalities judged to have high-quality vital information were included. Nevertheless, misclassification is not

For peer review only - http://bmjopen.bmj.com/site/about/guidelines.xhtml

expected to be a major concern because all deaths due to reported external causes, including homicides and suicides, are determined based on autopsy (19).

Results in Context

The results of this study are in contrast with a previous cross-sectional ecological study of the association between homicide and suicide rates in Brazil, which observed a significant inverse correlation between the two forms of death. In their study, Bando and Lester found a correlation of -0.61 ($p<0.001$) between age-standardised homicide rates and age-standardised suicide rates in 27 Brazilian states, and after stratification by sex, the correlation remained significant in men, but not women (16). Our unadjusted analysis using only the 2010 data showed similar results to those observed by Bando and Lester, however, after adjusting for confounders the association was no longer significant. Confounding factors appeared to have considerable influence on the observed relationship, and the inverse association in the unadjusted analysis is likely a reflection of bias. Further, it is notable that data for our analysis were aggregated to the municipality-level (n=5507), rather than state-level (n=27). Using more fine-grained geographical areas likely resulted in less heterogeneity within regions in terms of important determinants of homicide and suicide.

For peer review only

Our study found some differences in the association by age group. We found no association between increased homicide rates with increased suicide among those 10 to 19 years-old or 60 years and older. However, a statistically significant association was observed among men and women 20 to 59 years old. It is possible that the limited number of suicides among the youngest and oldest age-sex stratifications resulted in insufficient power to achieve significance. Nevertheless, this observation is consistent

For peer review only - http://bmjopen.bmj.com/site/about/guidelines.xhtml

with findings from a study of the correlations between homicide and suicide rates in Canada and US, which also showed the strongest correlations among 35-44 and 45-54 year-olds (28). Interestingly, there was nearly no difference in the strength of association between men and women within this age group, while both suicide and homicide rates tend to be higher in men (4,6). This finding aligns with other evidence that homicide and suicide rates in men and women tend to fluctuate similarly over time, despite differences in absolute rates, which may be because many predisposing factors for homicide and suicide are similar in men and women (29).

The relationship between homicide and suicide in the population has been studied for centuries and several theoretical frameworks for understanding their relationship have been suggested (13). Historically, homicide and suicide have been conceptualised as alternate expressions of the same underlying processes, therefore suggesting an inverse association between the two forms of death. For example, Henry and Short (1954) viewed them as alternate expressions of aggression (30). More recently, Unnithan and colleagues (1994) developed the *stream analogy*, that homicide and suicide are alternate forms of violence that depend on factors that indicate the amount of violence (forces of production) and whether the violence is expressed as homicide or suicide (forces of direction) (31). On the other hand, several others have questioned the inverse relationship, primarily arising from studies showing similar trends in homicide and suicide rates over time (32,33). For example, Holinger and Klemen (1982) studied mortality rates between 1900 and 1975 and found that rates of homicide, suicide and accidental death tended to fluctuate similarly over time (33). McKenna, Kelleher, and Corcoran (1997) also believed the relationship between homicide and suicide rates to be positive, reflecting the degree of social disorder in a population. Using data from the

For peer review only - http://bmjopen.bmj.com/site/about/guidelines.xhtml

Republic of Ireland and Northern Ireland between 1950 and 1990, they observed a positive correlation during times of peace, but an inverse correlation during a war-like period. Lester (1987) also questioned whether suicide and homicide are opposites based on findings from a literature review (34), despite interpreting earlier evidence in favour of Henry and Short's hypothesis (35).

The wide range of theories about the relationship reflects the highly contradictory findings of existing research. Studies based on multi-national data suggest differential relationships by country or geographical area. For example, using cross-sectional data from a large sample of UN member states for 1990-1999 compiled by WHO, Bills and Li (2005) examined the correlation between homicide and suicide rates within each region. The strength and direction of the relationship depended heavily on geographical region; a positive correlation between homicide and suicide rates was observed for Europe (r=0.89), whilst an inverse correlation was found for the Americas (r=-06.2) and Asian Pacific region (-0.97) (14). However, these results were not replicated in a later study by Fountoulakis et al. (2017), which used data from several countries between 2000 and 2010 that was also compiled by WHO (15).

Importantly, many of the existing studies have examined the association with limited control for potential confounding factors, which may help to explain the variations in correlation estimates observed between studies. Large areas, such as whole countries, likely contain considerable variability in the distributions of these important demographic and socioeconomic factors that influence homicide and suicide rates and estimates of their association. Furthermore, few studies have accounted for temporality, which may have an important influence on the association. Therefore, better evidence,

For peer review only - http://bmjopen.bmj.com/site/about/guidelines.xhtml

accounting for confounding factors, is required in order to draw clearer conclusions about the association beyond Brazil.

We observed a positive relationship between homicide and suicide rates that persisted even after adjusting for several important factors including per capita income, CAPS coverage, urbanisation, unemployment, single-person households, divorce, and religious affiliation. Therefore, we have evidence that, in Brazil, the relationship exists, even after accounting for these factors.

A positive relationship between homicide and suicide rates suggest that they may share some common social causes, yet no single theory appears to adequately explain the mechanism underlying this relationship. Statistically, a relationship may arise due to a causal association between homicide and suicide rates or due to shared causal factors. For example, high homicide rates may cause high suicide rates indirectly through low social cohesion within the geographical area under study. Alternatively, low social cohesion may cause high homicide rates and high suicide rates independently, therefore producing a spurious relationship. Two of these variables we considered—single-person households, and the percentage of individuals within the municipality that are divorced—may contribute to social cohesion. Since the relationship persists after adjusting for these, it is unlikely that low social cohesion adequately explains the relationship between homicide and suicide rates within the Brazilian municipalities. Notably, low social cohesion is only one possible explanation and there may be many others. Taken together, this is consistent with a hypothesis that changes in homicide rates lead to changes in suicide rates, though we cannot be confident that this reflects a

For peer review only - http://bmjopen.bmj.com/site/about/guidelines.xhtml

causal association. The relationship is likely complex, involving the interaction of social, cultural, economic, and political factors.

Targets of homicide and suicide prevention often have highly divergent orientations; while homicide prevention often involves the criminal justice system, suicide prevention usually involves the mental health system and reducing availability of methods for suicide. In Brazil, suicide is expected to be prevented at the community level of care. After the Psychiatric Reformation movement, mental health care in Brazil was officially reformulated in 1978, and the CAPS were created to provide mental health care in the community (36,37). However, increased CAPS coverage was not found to reduce total suicides in Brazilian municipalities between 2008 and 2012 (22). Likewise, to try to control violence the Brazilian justice system has increased incarceration, and as a result, Brazil has the fourth-largest prison population in the world (6), and homicide rates have increased between 2004 and 2012 (21). This highlights the importance of investigating new factors and potential targets for intervention and prevention that may be associated with suicide and homicide rates in the country.

The presence of a relationship between the two suggests the co-benefits of interventions to reduce homicide and suicide deaths. For example, studies in Brazil have shown an association between high coverage of the Brazilian cash transfer program (a social welfare program, which provides financial aid to low income Brazilians) and decreased municipal rate of suicide and homicide (21,22). In addition, extensive evidence from the US shows that restricting access to firearms has been shown to reduce firearm-related homicide and suicide (38). The correlation between firearm availability and suicide and

For peer review only - http://bmjopen.bmj.com/site/about/guidelines.xhtml

homicide appears to extend to other countries (39). In Brazil, a nationwide anti-firearm legislation was passed in 2004. There is some evidence that this legislation reduced both firearm-related suicide and firearm-related homicide, although the evidence for a reduction in total homicide and suicide is less clear (40,41). Unfortunately, the disarmament law was repealed in 2019. Future research may seek to identify other interventions at the policy level, which may help to reduce both homicide and suicide rates in the population.

CONCLUSION

Our longitudinal ecological study showed a robust positive relationship between homicide and suicide rates in Brazilian municipalities. We showed a dose-response relationship such that areas of higher homicide rates showed greater increase in suicide rates. The results of this study contribute clear evidence for the relationship to a literature that has previously shown conflicting evidence. A positive relationship between homicide and suicide rates can have important implications for public health and policy interventions as approaches to reduce one of homicide or suicide rates may also help to alleviate rates of the other. This can have important implications for Brazil and other countries where levels of violence are considerable.

CONTRIBUTORS

DM, LC, FA and MB were responsible for the conception of the study. DM obtained the data and conducted the data analysis. DM and KM contributed equally to the preparation of the manuscript. DM, KM, LC, DD, FA, LK, GL and MB contributed to the interpretation of results, provided critical review of the manuscript, and had final responsibility for the decision to submit for publication.

For peer review only - http://bmjopen.bmj.com/site/about/guidelines.xhtml

FUNDING

The study was in part funded by the Medical Research Council (MC_PC_MR/R018677/1) and Wellcome Trust (202912/Z/16/Z). This work was also supported by the National Institute for Health Research, University College London Hospital, Biomedical Research Centre. The funders were not involved in the design and conduct of the study; collection, management, analysis, and interpretation of the data; preparation, review, or approval of the manuscript; and decision to submit the manuscript for publication.

COMPETING INTERESTS

The authors report no competing interests.

PATIENT CONSENT

Consent was not required for this study, which involved secondary analysis of aggregated, anonymised data.

DATA SHARING

All data used in this study are publicly available online (http://www2.datasus.gov.br).

ETHICAL APPROVAL

This study used anonymized secondary data, therefore, ethical approval was not required.

For peer review only - http://bmjopen.bmj.com/site/about/guidelines.xhtml

REFERENCES

1. World Health Organization. Preventing suicide: A global imperative. Geneva, Switzerland, Switzerland: World Health Organization; 2014.

2. Nock MK, Borges G, Bromet EJ, Cha CB, Kessler RC, Lee S. Suicide and suicidal behavior. Epidemiol Rev. 2008;30(1):133–54.

3. Machado DB, Rasella D, Dos Santos DN. Impact of income inequality and other social determinants on suicide rate in Brazil. PLoS One. 2015;10(4):e0124934.

4. Martini M, da Fonseca RC, de Sousa MH, de Azambuja Farias C, Cardoso T de A, Kunz M, et al. Age and sex trends for suicide in Brazil between 2000 and 2016. Soc Psychiatry Psychiatr Epidemiol [Internet]. 2019;54(7):857–60. Available from: https://doi.org/10.1007/s00127-019-01689-8

5. World Health Organization. Preventing violence and reducing its impact: How development agencies can help. Geneva: Switzerland: World Health Organization; 2008.

6. Murray J, de Castro Cerqueira DR, Kahn T. Crime and violence in Brazil: Systematic review of time trends, prevalence rates and risk factors. Aggress Violent Behav. 2013;18(5):471–83.

7. Marinho F, de Azeredo Passos VM, Carvalho Malta D, Barboza França E, Abreu DMX, Araújo VEM, et al. Burden of disease in Brazil, 1990–2016: a systematic subnational analysis for the Global Burden of Disease Study 2016. Lancet. 2018;392(10149):760–75.

8. World Bank. GDP ranking [Internet]. 2017. Available from: https://datacatalog.worldbank.org/

9. World Bank. GINI index [Internet]. 2016. Available from: https://data.worldbank.org/indicator/SI.POV.GINI

For peer review only - http://bmjopen.bmj.com/site/about/guidelines.xhtml

10. Araújo EM de, Costa M da CN, Oliveira NF de, Santana F dos S, Barreto ML, Hogan V, et al. Spatial distribution of mortality by homicide and social inequalities according to race/skin color in an intra-urban Brazilian space. Rev Bras Epidemiol. 2010;13(4):549–60.

11. Gawryszewski VP, Costa LS. Social inequality and homicide rates in Sao Paulo City, Brazil. Rev Saude Publica. 2005;39(2):191–7.

12. Pereira DVS, Mota CMM, Andresen MA. Social disorganization and homicide in Recife, Brazil. Int J Offender Ther Comp Criminol. 2017;61(14):1570–92.

13. Bills CB. The Relationship between Homicide and Suicide: A narrative and conceptual review of violent death. Int J Confl Violence. 2017;11:a400–a400.

14. Bills CB, Li G. Correlating homicide and suicide. Int J Epidemiol. 2005;34(4):837–45.

15. Fountoulakis KN, Gonda X. Differential correlation of suicide and homicide rates according to geographical areas: A study with population-level data. Psychiatry Res. 2017;249:167–71.

16. Bando DH, Lester D. An ecological study on suicide and homicide in Brazil. Cien Saude Colet. 2014;19(4):1179–89.

17. World Health Organization. International statistical classification of diseases and related health problems: 10th revision ICD-10. Geneva: World Health Organization; 1992.

18. Fundação Nacional da Saúde. Manual de Instruções para o Preenchimento da Declaração de Óbito [Internet]. Brasília - Brazil: Ministério da Saúde; 2001. Available from: http://bvsms.saude.gov.br/bvs/publicacoes/manual_declaracao_obitos.pdf

19. Jorge MHP de M, Gotlieb SLD, Laurenti R. O sistema de informações sobre

For peer review only - http://bmjopen.bmj.com/site/about/guidelines.xhtml

mortalidade: problemas e propostas para o seu enfrentamento I - Mortes por causas naturais. Rev Bras Epidemiol. 2002;5(2):197–211.

20. Bertolote JM, Fleischmann A, De Leo D, Wasserman D. Psychiatric diagnoses and suicide: revisiting the evidence. Crisis. 2004;25(4):147–55.

21. Machado DB, Rodrigues LC, Rasella D, Barreto ML, Araya R. Conditional cash transfer programme: Impact on homicide rates and hospitalisations from violence in Brazil. PLoS One. 2018;13(12):e0208925.

22. Alves FJO, Machado DB, Barreto ML. Effect of the Brazilian cash transfer programme on suicide rates: a longitudinal analysis of the Brazilian municipalities. Soc Psychiatry Psychiatr Epidemiol. 2019;54(5):599–606.

23. Passos M, Zorzin PLG, Rocha D. O que (não) dizem os números – para além das estatísticas sobre o Novo Mapa Religioso Brasileiro (The meaning of numbers - beyond statistics on the Brazilian New Religious Map). DOI: 10.5752/P.2175-5841.2011v9n23p690. Horizonte. 2011;9(23).

24. Wooldridge JM. Introductory Econometrics: A Modern Approach. Econ Anal. 2003;

25. Hilbe JM. Negative binomial regression. Cambridge University Press; 2011.

26. Szwarcwald CL. Strategies for improving the monitoring of vital events in Brazil. Int J Epidemiol. 2008;37(4):738–44.

27. StataCorp. Stata Statistical Software: Release 15. College Station, TX, TX: StataCorp LP; 2017.

28. Leenaars AA, Lester D. Suicide and homicide rates in Canada and the United States. Suicide Life-Threatening Behav. 1994;24(2):184–91.

29. Batton C. Gender differences in lethal violence: Historical trends in the relationship between homicide and suicide rates, 1960-2000. Justice Quarterly.

For peer review only - http://bmjopen.bmj.com/site/about/guidelines.xhtml

2004.

30. Henry AF, Short JF. Suicide and homicide: Some econonmic, sociological and psychological aspects of aggression. New York: Free Press of Glencoe; 1954.

31. Unnithan P, Huff-Corzine L, Corzine J, Whitt HP. The Currents of Lethal Violence: An Integrated Model of Suicide and Homicide. Albany, NY: State University of New York Press; 1994.

32. Porterfield AL. Traffic Fatalities, Suicide, and Homicide. Am Sociol Rev [Internet]. 1960 Aug 27;25(6):897. Available from: http://www.jstor.org/stable/2089987

33. Holinger PC, Klemen EH. Violent deaths in the United States, 1900-1975. Relationships between suicide, homicide and accidental deaths. Soc Sci Med. 1982;16(22):1929–38.

34. Lester D. Murders and suicide: Are they polar opposites. Behav Sci Law. 1987;

35. Lester D. The association between the quality of life and suicide and homicide rates. J Soc Psychol. 1984;

36. Ministério da Saúde. Reforma Psiquiátrica e política de Saúde Mental no Brasil: 15 anos depois de Caracas [Internet]. 2005. Available from: http://portal.saude.gov.br/portal/arquivos/pdf/relatorio_15_anos_caracas.pdf

37. Ministério da Saúde. Portaria GM/MS 3088 [Internet]. Ministério da Saúde, editor. 2011. Available from: http://bvsms.saude.gov.br/bvs/saudelegis/gm/2011/prt3088_23_12_2011_rep.htm l

38. Rodríguez Andrés A, Hempstead K. Gun control and suicide: The impact of state firearm regulations in the United States, 1995-2004. Health Policy (New York). 2011;101(1):95–103.

For peer review only - http://bmjopen.bmj.com/site/about/guidelines.xhtml

39. Killias M, Van Kesteren J, Rindlisbacher M. Guns, violent crime, and suicide in 21 countries. Can J Criminol. 2001;43:429.

40. Cerqueira D, Mello JMP De. Evaluating a National Anti-Firearm Law and Etimating the Casual Effect of Guns on Crime [Internet]. Texto para discussão; 2017. Available from: https://econpapers.repec.org/RePEc:rio:texdis:607

41. de Fátima Marinho de Souza M, Macinko J, Alencar AP, Malta DC, de Morais Neto OL. Reductions In Firearm-Related Mortality And Hospitalizations In Brazil After Gun Control. Health Aff. 2007;26(2):575–84.

For peer review only

For peer review only - http://bmjopen.bmj.com/site/about/guidelines.xhtml

Supplementary Material

Machado DB, McDonald K, Castro-de-Araujo LFS, Devakumar D, Alves FJO, et al. **The association between homicide rates and suicide rates: a countrywide longitudinal analysis of 5,507 Brazilian municipalities.**

Contents

1. **Supplemental Methods**. Additional details about CAPS coverage
2. **Supplemental Figure 1.** Distribution of age-standardised suicide rates per 100,000.
3. **Supplemental Table 1.** Unadjusted and adjusted associations between age-standardized suicide and age-standardised homicide rates in all Brazilian municipalities, only municipalities with accurate vital information in Brazil, and by population size, 2008-2014.
4. **Supplemental Table 2**. Unadjusted and adjusted associations between suicide rates and homicide rates in all Brazilian municipalities for 2010 only.
5. **Supplemental References**

For peer review only

Supplemental Methods: Additional details about CAPS coverage

The Psychosocial Community Centers (CAPS) were developed to provide day hospital care severe mental disorders. There are five different types of CAPS, classified based on their complexity, population coverage, and funding. CAPS I are based in small towns (20,000-50,000 inhabitants), CAPS II are based in mid-sized cities (50,000 or more inhabitants), and CAPS III are large units intended to provide 24-hour services. CAPSi provide services to children and adolescents. CAPSad are specialised units to treat problems associated with drug and alcohol use. The Brazilian government estimates CAPS coverage within each municipality according to the proportion of the population served by the different types of units (1,2). Specifically, this is calculated as:
{[(number of CAPS I x 0.5) + (number of CAPS II) + (number of CAPS III x 1.5) + (number of CAPSi) + (number of CAPSad)] x 100,000} / municipal population

For peer review only

For peer review only - http://bmjopen.bmj.com/site/about/guidelines.xhtml

Supplemental Figure 1. Distribution of age-standardised suicide rates per 100,000.

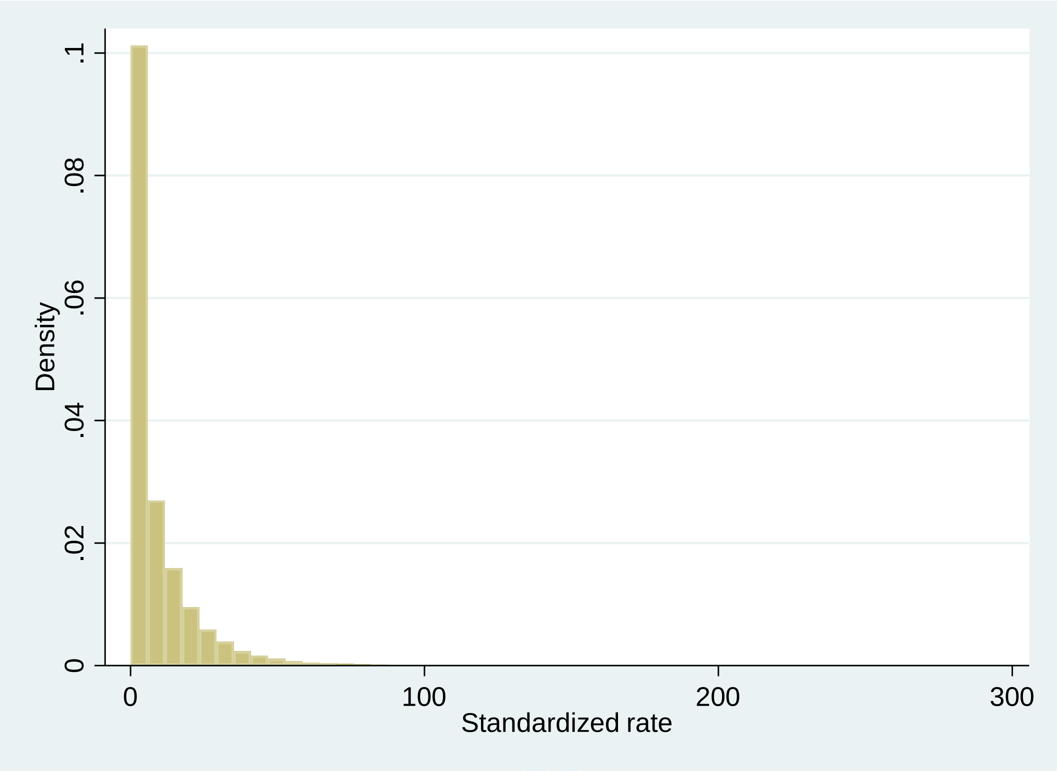

For peer review only - http://bmjopen.bmj.com/site/about/guidelines.xhtml

Supplemental Table 1. Unadjusted and adjusted associations between age-standardized suicide and age-standardised homicide rates in all Brazilian municipalities, only municipalities with accurate vital information in Brazil, and by population size, 2008-2014.

	**Unadjusted model**		**Adjusted model[a]**			
	**RR[b]**	**95% CI**	**RR[b]**	**95% CI**	**Number of suicides**	**Number of municipalities[c]**
All Brazilian municipalities	1.27	1.17-1.38	1.24	1.14-1.35	35,357	5,051
Municipalities with accurate vital information	1.46	1.23-1.72	1.45	1.22-1.72	9,336	1,556
Population <=10,000	1.21	1.03-1.41	1.21	1.03-1.41	14,352	2,100
Population 10,001-50,000	1.30	1.14-1.48	1.28	1.12-1.46	16,583	2,443
Population >50,000	1.33	1.15-1.55	1.32	1.13-1.54	4,259	639

Abbreviations: RR=Rate ratio; CI=Confidence interval.
[a] Models adjusted for monthly per capita income (BR$), CAPS coverage, urbanisation, percentage of people unemployed, percentage of individuals who were divorced, percentage of Pentecostal Christians, and percentage of households with one resident.
[b] Change in suicide rate per 100% increase in homicide rate.
[c] This number excludes municipalities with zero suicides recorded between 2008 and 2014.

For peer review only - http://bmjopen.bmj.com/site/about/guidelines.xhtml

Supplemental Table 2. Unadjusted and adjusted associations between suicide rates and homicide rates in all Brazilian municipalities for 2010 only.

	**Unadjusted model**		**Adjusted model[a]**			
**Year**	**RR[b]**	**95% CI**	**RR[b]**	**95% CI**	**Number of suicides**	**Number of municipalities[c]**
2010	0.51	0.46-0.64	0.91	0.77-1.08	9287	5,506

Abbreviations: RR=Rate ratio; CI=Confidence interval.
[a] Models adjusted for monthly per capita income (BR$), CAPS coverage, urbanisation, percentage of people unemployed, percentage of individuals who were divorced, percentage of Pentecostal Christians, and percentage of households with one resident.
[b] Change in suicide rate per 100% increase in homicide rate.
[c] This number excludes municipalities with zero suicides recorded in 2010.

For peer review only

Supplemental References

1. Ministério da Saúde. Portaria GM/MS 336 [Internet]. Ministério da Saúde, editor. 2002. Available from: http://www.saude.mg.gov.br/images/documentos/Portaria_336.pdf
2. Ministério da Saúde. Portaria GM/MS 2.669 [Internet]. Ministério da Saúde, editor. 2009. Available from: http://bvsms.saude.gov.br/bvs/saudelegis/gm/2009/prt2669_03_11_2009.html

For peer review only

For peer review only - http://bmjopen.bmj.com/site/about/guidelines.xhtml

STROBE Statement—checklist of items that should be included in reports of observational studies

	Item No	Recommendation	Page No
**Title and abstract**	1	(*a*) Indicate the study's design with a commonly used term in the title or the abstract<br><br>We conducted a longitudinal ecological study using annual mortality data from 5,507 Brazilian municipalities between 2008 and 2014.	2
		(*b*) Provide in the abstract an informative and balanced summary of what was done and what was found<br><br>**Objective:** To estimate the association between homicide rates and suicide rates in Brazilian municipalities over a period of 7 years.<br>**Design:** We conducted a longitudinal ecological study using annual mortality data from 5,507 Brazilian municipalities between 2008 and 2014. Multivariable negative binomial regression models were used to examine the relationship between homicide and suicide rates. Robustness of results was explored using sensitivity analyses to examine the influence of data quality, population size, age, and sex on the relationship between homicide and suicide rates.<br>**Setting:** A nationwide study of municipalities-level data.<br>**Participants:** Mortality data and corresponding population estimates for municipal populations aged 10 years and older.<br>**Primary and Secondary Outcome Measures:** Age-standardised suicide rates per 100,000.<br>**Results:** Municipal suicide rates were positively associated with municipal homicide rates; after adjusting for socioeconomic and demographic factors, a doubling of the homicide rate was associated with 22% increase in suicide rate (RR=1.22, 95% CI 1.26-1.33). A dose-response effect was observed with 4% increase in suicide rates at the third quintile, 9% at the fourth quintile and 12% at the highest quintile of homicide rates compared with the lowest quintile. The observed effect estimates were robust to sensitivity analyses.<br>**Conclusions:** Municipalities with higher homicide rates have higher suicide rates, and the relationship between homicide and suicide rates in Brazil exists independently of many sociodemographic and socioeconomic factors. The identification of a positive relationship between homicide and suicide rates suggests that population-based interventions to reduce homicide rates may also reduce suicide rates in Brazil.	2
**Introduction**			
Background/rationale	2	Explain the scientific background and rationale for the investigation being reported<br><br>Suicide is an important global public health problem, causing over 800,000 deaths per year….<br>In 2008, the World Health Organization (WHO) reported violence as a leading cause of death and disability and a "global challenge" (1)…	6-7

For peer review only

		While often studied separately, there is a long tradition of viewing homicide and suicide as related phenomena (13). Homicide and suicide can be viewed similarly as forms of violence (1). Several studies have examined the relationship between homicide and suicide rates in the population; however, evidence is mixed (see Research in Context panel). There appear to be strong variations in the magnitude and direction of association across countries (14,15). Many of the previous studies of the association have been conducted in HICs. However, the relationship between homicide and suicide rates in LMICs might be different due to different distributions and influences of risk factors in the population. There has been only one previous study of the ecological association between homicide and suicide rates in Brazil, which observed an inverse correlation in 2010 (16). However, this study used data from a single year, studied large, heterogeneous geographical areas (27 Brazilian states), and did not adjust for important demographic and socioeconomic factors.	
Objectives	3	State specific objectives, including any prespecified hypotheses<br><br>We aimed to further investigate the possible association between homicide and suicide rates in Brazil, using a dataset of all Brazilian municipalities from 2008 to 2014.	7
**Methods**			
Study design	4	Present key elements of study design early in the paper<br><br>This study used a longitudinal ecological design, using yearly data from the 5,507 Brazilian municipalities for the period 2008 to 2014.	7
Setting	5	Describe the setting, locations, and relevant dates, including periods of recruitment, exposure, follow-up, and data collection<br><br>See (4).	7
Participants	6	(*a*) *Cohort study*—Give the eligibility criteria, and the sources and methods of selection of participants. Describe methods of follow-up<br>*Case-control study*—Give the eligibility criteria, and the sources and methods of case ascertainment and control selection. Give the rationale for the choice of cases and controls<br>*Cross-sectional study*—Give the eligibility criteria, and the sources and methods of selection of participants<br><br>Ecological study design:<br><br>All data used in this study are publicly available online (http://www2.datasus.gov.br). To estimate suicide and homicide rates, cause-specific mortality data were collected from the Brazilian Ministry of Health's Mortality Information System. All deaths are recorded in this system using the International Classification of Diseases, 10th revision (17). Socioeconomic, demographic variables (monthly per capita income in Brazilian Real (BR$); unemployment rate; percentage of individuals who were divorced; percentage of individuals who declared being Pentecostal; percentage of households with only one resident; and urbanisation), and	8-9

For peer review only - http://bmjopen.bmj.com/site/about/guidelines.xhtml

		municipality population estimates were obtained from the Brazilian Institute of Geography and Statistics (IBGE). Data from the main mental health services in Brazil, the Psychosocial Care Centre (CAPS) coverage was obtained from the mental health coordination database, held by the Ministry of Health (DATASUS). …<br><br>Suicide is defined as a death resulting from intentional self-harm according to the International Classification of Diseases, 10th revision (ICD-10) codes X60 to X84 (17). …<br><br>Suicides among individuals under 10 years-old were excluded because suicide is a very rare event before 10 years; there were 33 victims of suicide under 10 years in Brazil in the period 2008-2014 (http://www2.datasus.gov.br). …<br><br>Homicide was defined as a death resulting from injuries inflicted by another person with the intent to injure or kill using any means according to the ICD-10 codes X85 to Y09 (17).	
		(*b*) *Cohort study*—For matched studies, give matching criteria and number of exposed and unexposed<br>*Case-control study*—For matched studies, give matching criteria and the number of controls per case	
Variables	7	Clearly define all outcomes, exposures, predictors, potential confounders, and effect modifiers. Give diagnostic criteria, if applicable<br><br>See (6). Also:<br><br>Municipality-level covariates for suicide considered in the model included: the monthly per capita income in Brazilian Real (BR$); unemployment rate, measured by the percentage of the population aged 16 years or over with no official job; percentage of individuals who were divorced; percentage of individuals who declared being Pentecostal; percentage of households with only one resident; and urbanisation, measured as the percentage of individuals living in urban areas for each municipality. These variables were selected based on evidence in the literature that they may be associated with suicide (3,20–22).	7-10
Data sources/ measurement	8*	For each variable of interest, give sources of data and details of methods of assessment (measurement). Describe comparability of assessment methods if there is more than one group<br><br>See (6) and (7).	7-10
Bias	9	Describe any efforts to address potential sources of bias<br><br>To examine the robustness of our primary findings, we fitted several additional negative binomial regression models: one including only municipalities considered to have very accurate vital information according to a previous study (25), three by total municipal population (10,000	12

For peer review only

		inhabitants; 10,001-50,000; and more than 50,000 inhabitants), one by municipal homicide rates quintiles, and six further stratified by sex and age groups (10-19 years; 20-59 years; 60 and older). Finally, we tested the association between homicide rates and age-standardised suicide rates for 2010 only to enable a comparison with the results of a previous study of Brazilian states (16). For each analysis, we fitted both unadjusted and adjusted models. Statistical analyses were performed using Stata 15 software (26).	
Study size	10	Explain how the study size was arrived at<br><br>Not applicable; the study was based on all municipalities in Brazil.	N/A

For peer review only

Quantitative variables	11	Explain how quantitative variables were handled in the analyses. If applicable, describe which groupings were chosen and why<br><br>See (7). Also Supplementary Methods and:<br>The main estimated regression model was: log(Yit) = αi + β1HOMICIDEit + βnXnit + γt + uit, where t =year and i =municipality, Yit is the standardized mortality rate (per 100,000 inhabitants) due to suicide, αi is the fixed effect for municipality i capturing unobserved time-invariant factors causing between-municipality variability in suicide, HOMICIDEit was the rate of homicide for the municipality i in the year t, Xnit is the value taken by a covariate vector containing the n socioeconomic and demographic determinants included in the model, for municipality i in year t, γt is a time-specific effect, capturing variability in suicide rates over time, uit is an error term, assumed to follow a normal distribution. To produce rate ratio (RR) estimates that were more readily interpretable, the homicide rate was divided by 100 (i.e. homicide rate per 1,000).<br><br>The age-standardised suicide rate, although not a count, still appeared to follow a binomial distribution (Supplemental Figure 1). We compared a negative binomial model to a Poisson regression model using an Akaike's Information Criterion and the Bayesian Information Criterion to establish the model that best fitted the data (24). We found evidence that the age-standardised suicide rates varied between municipalities more than expected under a Poisson distribution, therefore, the negative binomial model was used to allowed for overdispersion. The Hausman test was used to compare a negative binomial regression with fixed effects to a model with a random effect for each municipality. The test showed no evidence that the random effects model provided a better fit to the data (p=0.05), therefore, our model included only fixed effects.<br><br>Population standardization was used to allow comparability across municipalities with distinct age structures. We also tested the association using models based on both age-standard homicide rates and age-standardised suicide rates, and the results were similar (Supplemental Table 1).	7-10, Supplementary Materials, 10-11
Statistical methods	12	(*a*) Describe all statistical methods, including those used to control for confounding<br><br>See (7) and (11).. Also:<br>For each analysis, we fitted both unadjusted and adjusted models.	7-10, 11
		(*b*) Describe any methods used to examine subgroups and interactions<br><br>See (11).	11
		(*c*) Explain how missing data were addressed<br><br>Not applicable.	N/A
		(*d*) *Cohort study*—If applicable, explain how loss to follow-up was addressed	

For peer review only

Case-control study—If applicable, explain how matching of cases and controls was addressed<br>*Cross-sectional study*—If applicable, describe analytical methods taking account of sampling strategy	
(*e*) Describe any sensitivity analyses<br><br>See (9).	12

Continued on next page

For peer review only

Results

Participants	13*	(a) Report numbers of individuals at each stage of study—eg numbers potentially eligible, examined for eligibility, confirmed eligible, included in the study, completing follow-up, and analysed<br><br>Not applicable. The number of municipalities (n=5,506 are reported in the title, abstract and methods). Numbers of events (suicides) and municipalities in each analysis are reported in the tables/table legends.	N/A
		(b) Give reasons for non-participation at each stage<br><br>Not applicable.	N/A
		(c) Consider use of a flow diagram<br><br>Not applicable.	N/A
Descriptive data	14*	(a) Give characteristics of study participants (eg demographic, clinical, social) and information on exposures and potential confounders<br><br>See Table 1.	13
		(b) Indicate number of participants with missing data for each variable of interest<br><br>Not applicable.	N/A
		(c) *Cohort study*—Summarise follow-up time (eg, average and total amount)<br><br>Not applicable.	N/A
Outcome data	15*	*Cohort study*—Report numbers of outcome events or summary measures over time<br><br>See Tables 2-4.	14, 16-17
		Case-control study—Report numbers in each exposure category, or summary measures of exposure	
		Cross-sectional study—Report numbers of outcome events or summary measures	
Main results	16	(*a*) Give unadjusted estimates and, if applicable, confounder-adjusted estimates and their precision (eg, 95% confidence interval). Make clear which confounders were adjusted for and why they were included<br><br>See Tables 2-4. Columns of unadjusted and adjusted estimates are provided. Confounders are listed in the table legends.	14, 16-17
		(*b*) Report category boundaries when continuous variables were categorized	
		(*c*) If relevant, consider translating estimates of relative risk into absolute risk for a meaningful time period	
Other analyses	17	Report other analyses done—eg analyses of subgroups and interactions, and sensitivity analyses<br><br>Analysing by quintile of homicide rate showed that the higher the homicide rate, the stronger the association, with 1% suicide rate increase at the second quintile,	15-18

For peer review only - http://bmjopen.bmj.com/site/about/guidelines.xhtml

		4% increase at the third quintile, 9% increase at the fourth quintile, and 12% at the highest quintile compared with those in the lowest quintile (Table 3). The analysis stratified by sex and age groups showed a positive association between homicide rates and suicide among men from 20 to 59 years old (Table 4).<br><br>Table 3<br><br>Table 4<br><br>We repeated the analysis based on data from 2010 only. The unadjusted analysis showed a significant inverse relationship between homicide rates and suicide rates in 2010 (RR=0.51, 95% CI: 0.746-0.64). However, after adjusting for confounders the estimate of association became closer to the null value (RR=1.0) and was longer significant (RR=0.91, 95% CI: 0.77-1.08; Supplemental Table 2).	
**Discussion**			
Key results	18	Summarise key results with reference to study objectives<br><br>We assessed the relationship between homicide and suicide rates in Brazilian municipalities over a 7-year period. We found that municipalities with higher homicide rates also had higher age-standardised suicide rates in both unadjusted and adjusted models. Our results appeared to be robust based on several sensitivity analyses. Similar results were observed for models stratified by population size, and for models including only municipalities considered to have accurate data (25). The association between homicide and suicide rates seemed to follow a dose-response pattern, such that areas with higher homicide rates showed stronger relationships with increased suicide rates, reaching 12% increase in the highest quintile of homicide rates compared with the lowest.	18
Limitations	19	Discuss limitations of the study, taking into account sources of potential bias or imprecision. Discuss both direction and magnitude of any potential bias<br><br>This study used an ecological design, and therefore conclusions are applicable to populations rather than individuals. An ecological design was used due to data availability, and only the use of aggregate data allows the study of the total Brazilian population longitudinally. Furthermore, given our interest in homicide rates as an indicator of contextual violence, aggregate data for homicide were required.<br><br>Another limitation could be that large municipalities and small municipalities have equal weight in the analysis. For this reason, we have also provided estimates after stratification by municipal population size.<br><br>Potential bias resulting from misclassification of suicides and homicides is another possible limitation. Homicide and suicide deaths may have a risk of misclassification as events of undetermined intent or unspecified accidents. Suicide may be underreported due to stigmatisation and social taboo, while homicides could be under-reported as they may indicate unrest or instability. It is unclear whether the frequency of misclassification differs between homicides and suicides. If misclassification of homicides and suicides occurred equally	18-19

For peer review only - http://bmjopen.bmj.com/site/about/guidelines.xhtml

For peer review only

		frequently, then the observed estimates of association between homicide and suicide rates are conservative. In fact, the observed estimate of association was slightly stronger when only municipalities judged to have high-quality vital information were included. Nevertheless, misclassification is not expected to be a major concern because all deaths due to reported external causes, including homicides and suicides, are determined based on autopsy (19).	
Interpretation	20	Give a cautious overall interpretation of results considering objectives, limitations, multiplicity of analyses, results from similar studies, and other relevant evidence<br><br>The results of this study are in contrast with a previous cross-sectional ecological study of the association between homicide and suicide rates in Brazil, which observed a significant inverse correlation between the two forms of death. …<br><br>In the broader literature, existing studies of the relationship between homicide and suicide rates provide mixed evidence. …<br><br>We observed that the relationship between homicide and suicide rates persisted even after adjusting for several important factors including per capita income, CAPS coverage, urbanisation, unemployment, single-person households, divorce, and religious affiliation. Therefore, we have evidence that, in Brazil, the relationship exists, even after accounting for these factors.	20-22
Generalisability	21	Discuss the generalisability (external validity) of the study results<br><br>We observed that the relationship between homicide and suicide rates persisted even after adjusting for several important factors including per capita income, CAPS coverage, urbanisation, unemployment, single-person households, divorce, and religious affiliation. Therefore, we have evidence that, in Brazil, the relationship exists, even after accounting for these factors.	22
**Other information**			
Funding	22	Give the source of funding and the role of the funders for the present study and, if applicable, for the original study on which the present article is based<br><br>See Funding.	24

*Give information separately for cases and controls in case-control studies and, if applicable, for exposed and unexposed groups in cohort and cross-sectional studies.

Note: An Explanation and Elaboration article discusses each checklist item and gives methodological background and published examples of transparent reporting. The STROBE checklist is best used in conjunction with this article (freely available on the Web sites of PLoS Medicine at http://www.plosmedicine.org/, Annals of Internal Medicine at http://www.annals.org/, and Epidemiology at http://www.epidem.com/). Information on the STROBE Initiative is available at www.strobe-statement.org.

For peer review only
